# Supplementary material for: The selection force weakens with age because ageing evolves and not vice versa
Source: Nat Commun. 2022 Feb 3;13:686. doi: 10.1038/s41467-022-28254-3 (PMC8813929; doi:10.1038/s41467-022-28254-3)
Supplement: Supplementary file 1 — Supplementary Information [file 41467_2022_28254_MOESM1_ESM.pdf]

The selection force weakens with age because ageing evolves and not vice versa

Supplementary Methods

Stefano Giaimo and Arne Traulsen

[1]Department of Evolutionary Theory, Max Planck Institute for Evolutionary Biology
August-Thienemann-Straße 2, 24306 Plön, Germany
giaimo@evolbio.mpg.de

Contents

1 Model, basic notation and definitions **3**
1.1 Demography . . . 3
1.1.1 Euler-Lotka equation . . . 3
1.1.2 Net reproduction rate, generation time and reproductive value . . . 3
1.2 Ageing . . . 4
1.3 Metric . . . 4
1.4 Fitness . . . 5
1.5 Selection gradients . . . 6
1.5.1 Selection gradients in the classic theory . . . 6
1.5.2 Generalized selection gradients . . . 7
1.6 Dynamics . . . 8
1.6.1 Dynamics of fecundity . . . 8
1.6.2 Dynamics of survival . . . 9

2 Analysis of fecundity evolution **9**
2.1 Equilibria in fecundity evolution . . . 9
2.2 Equilibrium selection gradient on fecundity . . . 10
2.3 Sets of functions for fecundity evolution . . . 10
2.3.1 Definition of $\mathscr{F}_+^m$ . . . 10
2.3.2 Definition of $\mathscr{F}_-^m$ . . . 11
2.4 Fecundity at equilibrium . . . 11
2.4.1 Ageing in fecundity at equilibrium . . . 11
2.4.2 Negative ageing in fecundity at equilibrium . . . 12
2.4.3 Uniqueness . . . 13
2.5 Dynamical properties of fecundity evolution . . . 13
2.5.1 Coordinate transformation . . . 13
2.5.2 Dynamics of the transformed system . . . 14
2.5.3 Basic properties of the transformed system . . . 15
2.5.4 Set stability of fecundity . . . 16

2.5.5 Stability of ageing in fecundity — Power functions . . . 19
2.5.6 Stability of ageing in fecundity — Exponential functions . . . 20
2.5.7 Instability of negative ageing in fecundity . . . 22
2.6 Numerical solutions . . . 24

3 Analysis of survival evolution **24**
3.1 Equilibria in survival evolution . . . 25
3.2 Equilibrium selection gradient on survival . . . 25
3.3 Sets of functions for survival evolution . . . 26
3.3.1 Definition of $\mathscr{F}_+^p$ . . . 26
3.3.2 Definition of $\mathscr{F}_-^p$ . . . 26
3.4 Survival at equilibria . . . 27
3.4.1 Ageing in survival at equilibrium . . . 27
3.4.2 Negative ageing in survival at equilibrium . . . 28
3.5 Dynamical properties of survival evolution . . . 28
3.5.1 Coordinate transformation . . . 28
3.5.2 Dynamics of the transformed system . . . 29
3.5.3 Basic properties of the transformed system . . . 29
3.5.4 Stability of survival to pre-reproductive ages . . . 31
3.5.5 Set stability of survival at reproductive ages . . . 34
3.6 Minimal model . . . 38
3.6.1 Equilibria and stability in the minimal model . . . 39
3.7 Boundedness . . . 45
3.8 Numerical solutions . . . 45

1 Model, basic notation and definitions

1.1 Demography

Basic demographic quantities that are used throughout are reviewed here. Notation largely follows [1].

1.1.1 Euler-Lotka equation

Let m_j and p_j be average fecundity and average survival, respectively, at individual age j in the population. The fraction of newborns surviving at least up to age class j is

$$l_j = p_0 p_1 p_2 ... p_{j-1}, \qquad j = 0, 1, ... \tag{SI.1}$$

with $l_0 = 1$. Reproduction begins at age α, i.e. $m_j = 0$, $j = 1, \ldots, \alpha - 1$, and is uninterrupted throughout, i.e. $m_j > 0$, $j = \alpha, \alpha + 1, \ldots$. To exclude the impossibility of death, survival is assumed throughout to be never "perfect" so that $p_j < 1$, $j = 0, 1, \ldots$. Holding reproduction and survival fixed, the population eventually attains a stable demographic equilibrium where the fraction in each age class is constant [1]. At this demographic equilibrium, the Euler-Lotka equation

$$\sum_{j=1}^{\infty} l_j m_j \lambda^{-j} = 1 \tag{SI.2}$$

determines the constant growth rate of the population, λ per time step . Of our interest are demographically stable populations that are also demographically stationary, i.e. size stays constant and $\lambda = 1$.

1.1.2 Net reproduction rate, generation time and reproductive value

The net reproduction rate

$$R = \sum_{j=1}^{\infty} l_j m_j \tag{SI.3}$$

gives the expected lifetime offspring production. In a demographically stationary population, one has $R = 1$ as reproduction is exactly at replacement level. The generation time (i.e. average age of parents) at demographic stability in the population is [1]

$$T = \sum_{j=1}^{\infty} j l_j m_j \lambda^{-j}. \tag{SI.4}$$

The reproductive value v_j at age j is

$$v_j = \frac{\lambda^j}{l_j} \sum_{i=j}^{\infty} l_i m_i \lambda^{-i}, \tag{SI.5}$$

which gives the residual expected number of offspring to an individual aged j where future offspring are discounted by population growth [1, 2]. The reproductive value at birth (yet before the individual is censused for the first time at its first birthday) is

$$v_0 = \sum_{i=1}^{\infty} l_i m_i \lambda^{-i} \tag{SI.6}$$

as defined in [1]. By Eq. (SI.2), in a demographically stable, selectively neutral population $v_0 = 1$.

1.2 Ageing

Here, the operational definitions of ageing and negative ageing that are adopted in the present work are given. These definitions are meant to apply to life histories with multiple reproductive events, i.e. iteroparity [3]. A unique reproductive event followed by death, i.e. semelparity, can be thought of as the most extreme form of ageing both in fecundity and in survival. Semelparity is not analyzed here. We focus on iteroparity. We follow the convention of regarding ageing as pertinent to adult life and we adopt the following conventions:

Ageing in fecundity:	$m_j > m_{j+1}$,	$j = \alpha, \alpha+1, \ldots$
Negative ageing in fecundity:	$m_j < m_{j+1}$,	$j = \alpha, \alpha+1, \ldots$
Ageing in survival:	$p_j > p_{j+1}$,	$j = \alpha-1, \alpha, \ldots$
Negative ageing in survival:	$p_j < p_{j+1}$,	$j = \alpha-1, \alpha, \ldots$

1.3 Metric

As we consider infinite ages, we will mostly deal with the space of sequences $(a_n) = a_1, a_2, \ldots$ of real numbers. These sequences will represent fecundity, survival or some transformation thereof at each age. Sometimes, it will be useful to retrieve the supremum, e.g. $\sup(a_n)$, or the infimum, e.g. $\inf(a_n)$, of such sequences. The space of sequences can be equipped with a metric $d((a_n),(b_n))$, which is a function that measures the distance between any two members (a_n) and (b_n) of this space, to obtain a metric space. For this purpose, we will use the metric:

$$d_i((a_n),(b_n)) = \sum_{j=i}^{\infty} \frac{1}{2^j} \frac{|a_j - b_j|}{1 + |a_j - b_j|}. \tag{SI.7}$$

The reason for letting open the choice for the starting index in the series above is that we will generally need to retrieve the distance between specific tails of sequences, as it will be clear during our exposition.

To show that d_i is a metric, we note that the series it gives rise to is convergent because the series

$$\sum_{j=0}^{\infty} \frac{1}{2^j} = 2 \tag{SI.8}$$

is convergent and is either equal to or larger than $d_i((a_n),(b_n))$. Clearly, the series in Eq. (SI.7) only contains non-negative terms and, therefore, it converges to a non-negative number. It converges to 0 if and only if $a_i = b_i$ for all terms in the series. We also have symmetry, i.e. $d_i((a_n),(b_n)) = d_i((b_n),(a_n))$, because $|a_j - b_j| = |b_j - a_j|$. Finally, d_i obeys the triangle inequality, i.e. $d_i((a_n),(b_n)) \leq d_i((a_n),(c_n)) + d_i((c_n),(b_n))$ for any sequence (c_n). This can be shown term-wise by noting that the function $z/(1+z)$ has first derivative $1/(1+z)^2$, which exists and is positive for $z > -1/2$. Therefore, this function is strictly increasing on $(-1/2, \infty)$ and preserves order on this interval. Using the triangle inequality for absolute value and order preservation, we have for the j-th term of d_i that

$$\begin{aligned} |a_j - b_j| \leq |a_j - c_j| + |c_j - b_j| &\Rightarrow \frac{|a_j - b_j|}{1 + |a_j - b_j|} \leq \frac{|a_j - c_j| + |c_j - b_j|}{1 + |a_j - c_j| + |c_j - b_j|} \\ &\Rightarrow \frac{|a_j - b_j|}{1 + |a_j - b_j|} \leq \frac{|a_j - c_j|}{1 + |a_j - c_j|} + \frac{|c_j - b_j|}{1 + |c_j - b_j|}. \end{aligned} \tag{SI.9}$$

As it is customary for metric spaces, the distance $d(z, A)$ between a member z and a non-empty subset A of the metric space is

$$d(z, A) = \inf_{a \in A} d(z, a), \tag{SI.10}$$

where the infimum is taken over all members a of A.

1.4 Fitness

Here, the fitness function to be used in the breeder's equation, Eq. (5) in the main text (Methods Section), is discussed. Two main approximations have been proposed in the literature:

(i) One is $\bar{w} \approx \ln\lambda$, which leads to

$$\frac{\partial \bar{w}}{\partial \bar{z}_j} \approx \frac{\partial \ln\lambda}{\partial \bar{z}_j}, \tag{SI.11}$$

which holds for age-structured populations assuming frequency and density independent selection and either permanent demographic equilibrium and multivariate normal joint distribution of traits [4], or small genetic covariances without the requirement of permanent demographic stability [5]. To keep the population of constant size (i.e. demographically stationary with $\lambda = 1$) while preserving the approximation in Eq. (SI.11), some form of density dependence is required. To study survival evolution, we will implement density dependence by scaling all survival probabilities with a constant factor, as suggested by [6] (more on this in Section 1.6).

(ii) The other approximation is obtained by equating fitness in the breeder's equation with the net reproductive rate R in Eq. (SI.3) so that we have

$$\frac{\partial \bar{w}}{\partial \bar{z}_j} \approx \frac{1}{T}\frac{\partial R}{\partial \bar{z}_j}, \tag{SI.12}$$

where T is obtained by setting $\lambda = 1$ in Eq. (SI.4), see [7]. As R measures population change per generation and not per time unit [8], an additional scaling by generation time is required. This approximation will be used to study fecundity evolution. In this case, to implement density dependence we scale all fecundities by a factor such that $R = 1$, as suggested by [6] (more on this in Section 1.6).

Next, we show that these two approaches lead to the same more general assumption. As only the first partial derivatives of fitness matter for present purposes, let φ be some parameter on which fecundity and survival depend. From implicit differentiation of Eq. (SI.2), we obtain

$$\frac{\partial \ln\lambda}{\partial \varphi} = \frac{1}{T}\frac{\partial}{\partial \varphi}\left(\sum_{i=1}^{\infty} l_i(\varphi) m_i(\varphi)\lambda^{-i}\right) \tag{SI.13}$$

and from direct differentiation of Eq. (SI.3) and division by the generation time, we find

$$\frac{1}{T}\frac{\partial R}{\partial \varphi} = \frac{1}{T}\frac{\partial}{\partial \varphi}\left(\sum_{i=1}^{\infty} l_i(\varphi) m_i(\varphi)\right). \tag{SI.14}$$

By differentiating the reproductive value at birth v_0 in Eq. (SI.6) in a stationary population with respect to the same parameter and dividing by the generation time while treating λ as a constant, we obtain

$$\frac{1}{T}\frac{\partial v_0}{\partial \varphi} = \frac{1}{T}\frac{\partial}{\partial \varphi}\left(\sum_{i=1}^{\infty} l_i(\varphi) m_i(\varphi)\lambda^{-i}\right). \tag{SI.15}$$

As only demographically stationary populations are considered, by setting $\lambda = 1$ the right hand sides of Eqs. (SI.13), (SI.14) and (SI.15) are identical. Thus, instead of using two different versions of Eq. (1) in the main text, a convenient and unified way of expressing the breeder's equation for demographically-stationary age-structured populations is

$$\bar{z}_j(t+1) = \bar{z}_j(t) + g_j \frac{1}{T(t)}\frac{\partial v_0}{\partial \bar{z}_j}(t)\bigg|_{\lambda=1} + \sum_{i\neq j} g_{ji}\frac{1}{T(t)}\frac{\partial v_0}{\partial \bar{z}_i}(t)\bigg|_{\lambda=1}. \tag{SI.16}$$

The use of reproductive value at birth as a measure of fitness is not new and is consistent with suggestions in [2, 4, 9, 1, 10]. This also explains our definition of fitness in Eq. (1) of the main text (Methods Section). In what follows, the gradient of reproductive value at birth is assumed to be taken in a stationary population. As stated in the main text, all trade-offs are removed by setting $g_{ji} = 0$ for $i \neq j$ and $g_j = \delta$, where $0 < \delta \ll 1$ so that the additive genetic variance is set equal for each trait at all time so that all traits are permanently subject to weak selection at the same intensity and they do not covary. Traits then evolve according to the breeder's equation in the form

$$\bar{z}_j(t+1) = \bar{z}_j(t) + \delta \frac{1}{T(t)} \frac{\partial v_0}{\partial \bar{z}_j}(t)\bigg|_{\lambda=1}. \tag{SI.17}$$

In what follows, the assumption of weak selection is used repeatedly to take linear approximations so that, in general, equations are valid to the first order in δ. Three things should be noted. First, the mechanisms of ecologically-induced density dependence discussed above (more on this in Section 1.6) have the effect of limiting fecundity and survival values. In what follows, the traits under study are fecundity and survival. As selection for fecundity and survival is always positive and trade-offs are assumed absent, fecundity and survival (and, therefore, population growth) would increase without bound in the absence of ecological pressure. Density dependence is then needed and it is assumed in parts of the classic theory as well [11, p. 36], which is also concerned with unconstrained selection of fecundity and survival. That the density dependent case is the most relevant and realistic was later recognized by Hamilton [12, p. 89] himself. Second, by assuming stationary populations, the model sets a condition to the evolution of ageing that is less favourable than that of an increasing population. One offspring produced later in life is a smaller contribution to an increasing population than one offspring produced earlier because the former offspring finds itself in a bigger population. Therefore, the slower the growth, the less this discounts future offspring value. Third, although the breeder's equation typically presupposes a finite number of traits, in what follows we will not generally make such restriction. We will consider age-specific fecundity and survival assuming potentially infinite ages and use Eq. (SI.17) with index j indicating age and running to infinity. In a few cases, however, it will be clearly stated that some results and, mainly, numerical simulations are obtained by placing a maximum age ω beyond which survival is not possible, i.e. $p_\omega(t) = 0$ for all time points t.

1.5 Selection gradients

Here, expressions for the gradient of v_0 on fecundity and survival are given. These expressions are general enough to accommodate any form of age-specific genetic variation. To facilitate comparison with previous work, these selection gradients are derived from the gradients found in the classic theory [11].

1.5.1 Selection gradients in the classic theory

Classic theory studies fecundity evolution by considering the slope of $\ln \lambda$ with respect to m_j [11]. Here, however, given the assumption of a stationary population, we look at the (proportionally equivalent) gradient of the net reproduction rate,

$$\frac{\partial R}{\partial m_j} = l_j > 0, \qquad j = \alpha, \alpha+1, \ldots \tag{SI.18}$$

which, by Eq. (SI.1), has the property that

$$\frac{\partial R}{\partial m_{j+1}} \bigg/ \frac{\partial R}{\partial m_j} = \frac{l_{j+1}}{l_j} = p_j < 1, \qquad j = \alpha, \alpha+1, \ldots \tag{SI.19}$$

i.e. positive selection on fecundity decreases with age. This property also pertains to the original gradient of the classic theory under the sufficient condition that $\lambda \geq 1$, i.e. the population is not going extinct.

In the classic life history theory of the evolution of ageing [11], survival evolution is studied by considering the gradient

$$H_j := \frac{\partial \ln \lambda}{\partial \ln p_j} = \frac{1}{T} \sum_{i=j+1}^{\infty} l_i m_i \lambda^{-i} > 0, \qquad j = 0, 1, \ldots \tag{SI.20}$$

which is obtained by implicit differentiation of Eq. (SI.2). Convergence of the series in Eq. (SI.20) is implied by convergence of the Euler-Lotka equation. This gradient has the properties

$$0 < H_j \leq 1, \qquad j = 0, 1, \ldots, \alpha - 1 \tag{SI.21a}$$
$$0 < H_j < 1, \qquad j = \alpha, \alpha + 1, \ldots \tag{SI.21b}$$
$$\frac{H_{j+1}}{H_j} = 1, \qquad j = 0, 1, \ldots, \alpha - 2 \tag{SI.21c}$$
$$0 < \frac{H_{j+1}}{H_j} < 1, \qquad j = \alpha - 1, \alpha, \ldots, \tag{SI.21d}$$

i.e. constant positive selection at pre-reproductive ages and decreasing selection with reproductive age. These properties are independent of the actual values of p_j and m_j and, therefore, of λ. By Eq. SI.21, the sequence $(H_n) = H_0, H_1, \ldots$ has terms that get progressively smaller for $j = \alpha, \alpha + 1, \ldots$ and are bounded below by 0. Therefore, by monotone convergence, $\lim(H_n) = 0$. Moreover, using Eq. (SI.4), it can be shown that the series $\sum_{j=0}^{\infty} H_j$ converges,

$$\sum_{j=0}^{\infty} H_j = \frac{1}{T} \sum_{j=0}^{\infty} \sum_{i=j+1}^{\infty} l_i m_i \lambda^{-i} = \frac{1}{T} \sum_{j=1}^{\infty} j l_j m_j \lambda^{-j} = \frac{T}{T} = 1, \tag{SI.22}$$

where the double series was reduced to one using

Age 1	Age 2	Age 3	Age 4	...	Age n	...
$l_1 m_1 \lambda^{-1}$	$l_2 m_2 \lambda^{-2}$	$l_3 m_3 \lambda^{-3}$	$l_4 m_4 \lambda^{-4}$	...	$l_n m_n \lambda^{-n}$	...
	$l_2 m_2 \lambda^{-2}$	$l_3 m_3 \lambda^{-3}$	$l_4 m_4 \lambda^{-4}$	...	$l_n m_n \lambda^{-n}$	...
		$l_3 m_3 \lambda^{-3}$	$l_4 m_4 \lambda^{-4}$	...	$l_n m_n \lambda^{-n}$	...
			$l_4 m_4 \lambda^{-4}$	...	$l_n m_n \lambda^{-n}$	...
				$\ddots$	$\vdots$	...
					$l_n m_n \lambda^{-n}$	...

by taking column sums instead of row sums.

1.5.2 Generalized selection gradients

Life history evolution may not only proceed by small additive changes in fecundity m_j and small multiplicative changes in survival p_j [13, 14], as assumed in the classic theory [11]. To capture this situation in a general way, let f_{M} and f_{P} be functions for which both the first derivative and the second derivative exist and are continuous. Furthermore, it is assumed throughout that

$$0 < f'_{\mathrm{M}}(m) < \infty, \quad m > 0 \tag{SI.23a}$$
$$0 < f'_{\mathrm{P}}(p) < \infty, \quad p \in (0, 1) \tag{SI.23b}$$

i.e. both f_{M} and f_{P} preserve order and have inverses f_{M}^{-1} and f_{P}^{-1}, respectively, in the relevant intervals. Suppose genetic variation is additive on $f_{\mathrm{M}}(m_j)$ for age-specific fecundity and on $f_{\mathrm{P}}(p_j)$ for age-specific survival. Using the chain rule, one gets

$$\frac{\partial R}{\partial f_{\mathrm{M}}(m_j)} = \frac{\partial m_j}{\partial f_{\mathrm{M}}(m_j)} \frac{\partial R}{\partial m_j} = \frac{l_j}{f'_{\mathrm{M}}(m_j)} > 0, \qquad j = \alpha, \alpha + 1, \ldots \tag{SI.24a}$$
$$\frac{\partial \ln \lambda}{\partial f_{\mathrm{P}}(p_j)} = \frac{\partial p_j}{\partial f_{\mathrm{P}}(p_j)} \frac{\partial \ln p_j}{\partial p_j} \frac{\partial \ln \lambda}{\partial \ln p_j} = \frac{1}{f'_{\mathrm{P}}(p_j)} \frac{H_j}{p_j} > 0, \qquad j = 0, 1, \ldots \tag{SI.24b}$$

As stationary populations are considered and Eq. (SI.17) is used, one can write

$$\left.\frac{1}{T}\frac{\partial v_0}{\partial f_{\mathrm{M}}(m_j)}\right|_{\lambda=1} = \left.\frac{1}{T}\frac{l_j}{f'_{\mathrm{M}}(m_j)}\right|_{\lambda=1}, \qquad j = \alpha, \alpha+1, \ldots, \tag{SI.25a}$$

$$\left.\frac{1}{T}\frac{\partial v_0}{\partial f_{\mathrm{P}}(p_j)}\right|_{\lambda=1} = \left.\frac{1}{f'_{\mathrm{P}}(p_j)}\frac{H_j}{p_j}\right|_{\lambda=1}, \qquad j = 0, 1, \ldots \tag{SI.25b}$$

which are generalized selection gradients. These expressions, which are derived from Eqs. (SI.13-SI.15) and Eq. (SI.24), give the components of the gradient of v_0 (divided by the generation time) that appear in our general form of the breeder's equation Eq. (SI.17) and are necessary to study fecundity evolution and survival evolution. To reduce clumsiness, the sign of evaluation at $\lambda = 1$ is dropped from now on.

The assumptions in Eq. (SI.23) ensure that selection favours fecundity and survival, because to an increase in $f_{\mathrm{M}}(m_j)$ there corresponds an increase in m_j and vice versa. The same holds for survival. Selection gradients point to increased fitness, considering selection against reduced fecundity or mortality would merely add the complication of a sign change.

1.6 Dynamics

The breeder's equation in Eq. (SI.17) updates the vector $\bar{\mathbf{z}}$ of mean traits in the population in discrete time steps. Here this equation is combined with the expressions in Eq. (SI.25) and with some form of density dependence as anticipated in Section 1.4. The resulting equations describe the evolution of a population of very large, constant size that is subject to selection acting on age-specific traits. The dynamics of fecundity evolution and the dynamics of survival evolution are considered separately.

1.6.1 Dynamics of fecundity

Here, the equations governing the evolution of fecundity m_j, $j = \alpha, \alpha+1, \ldots$, are given. In Eq. (SI.17), we set $\bar{z}_j(t) = f_{\mathrm{M}}(m_j(t))$ with $j = \alpha, \alpha+1, \ldots$. Survival values are assumed to form a sequence $p_0, p_1, \ldots$ of arbitrary, fixed constants each independently chosen from the interval $(0, 1)$. Let $m_j(0)$, $j = \alpha, \alpha+1, \ldots$ be the initial fecundities, which are all positive and such that $R(0) = 1$. Fecundities at pre-reproductive ages are set constant at zero throughout, i.e. $m_j(t) = \bar{z}_j(t) = 0$, $j = 1, \ldots, \alpha - 1$. Let

$$\hat{m}_j := f_{\mathrm{M}}^{-1}\left(f_{\mathrm{M}}(m_j) + \delta\frac{1}{T}\frac{l_j}{f'_{\mathrm{M}}(m_j)}\right), \qquad j = \alpha, \alpha+1, \ldots, \tag{SI.26}$$

be fecundity just after one iteration of the breeder's equation, yet before the next time step. Let $\Delta_{\mathrm{s}}R > 0$ be the corresponding increase in the net reproduction rate so that $1 + \Delta_{\mathrm{s}}R$ is the net reproduction rate in a population where fecundity at age $j = \alpha, \alpha+1, \ldots$ is fixed at $\hat{m}_j$. Dynamics for fecundity evolution are given by the equations,

$$m_j(t+1) = \frac{f_{\mathrm{M}}^{-1}\left(f(m_j(t)) + \delta\frac{1}{T(t)}\frac{l_j}{f'_{\mathrm{M}}(m_j(t))}\right)}{1 + \Delta_{\mathrm{s}}R(t)}, \qquad j = \alpha, \alpha+1, \ldots, \tag{SI.27}$$

where the denominator is

$$1 + \Delta_{\mathrm{s}}R(t) = \sum_{i=1}^{\infty} l_i \hat{m}_i(t), \tag{SI.28}$$

where, because of weak selection ($0 < \delta \ll 1$), the quantity $\Delta_{\mathrm{s}}R(t)$ is assumed small and the series on the right hand side convergent. The quantity in Eq. (SI.28) imposes ecological equilibrium by scaling fecundity at all ages by the same factor, see Section 1.4. As a result, $R(t) = 1$ for all t. Note that the dynamics in Eq. (SI.27) ensure that fecundity is always positive. Since f_{M} is strictly increasing in its argument and the selection gradient on fecundity is always positive, the numerator in Eq. (SI.27) at t is always greater than $m_j(t)$. The denominator is always positive. Hence, at each time step, age-specific fecundity is first increased and, then, scaled by a positive quantity thereby keeping fecundity positive independently of t.

1.6.2 Dynamics of survival

Here the equations governing the evolution of survival p_j, $j = 0, 1, \ldots$, are given. In Eq. (SI.17), we set $\bar{z}_j(t) = f_{\mathrm{P}}(p_j(t))$. Fecundities m_j are assumed to form a sequence $0, 0, \ldots, 0, m_\alpha, m_{\alpha+1}, \ldots$ of arbitrary finite constants independent of t that are zero at pre-reproductive ages and positive at reproductive ages. Let $p_j(0)$, $j = 0, 1, \ldots$ be the initial survival values. These are chosen in the $(0, 1)$ interval independently of one another. The corresponding survivorship, see Eq. (SI.1), is $l_j(0)$, $j = 0, 1, \ldots$. Initial survival values are such that $\lambda = 1$ solves the Euler-Lotka equation for the given fecundities so that

$$\sum_{j=1}^{\infty} l_j(0) m_j = 1. \tag{SI.29}$$

Let then

$$\hat{p}_j := f_{\mathrm{P}}^{-1}\left(f_{\mathrm{P}}(p_j) + \delta \frac{1}{T} \frac{\partial v_0}{\partial f_{\mathrm{P}}(p_j)}\right), \qquad j = 0, 1 \ldots, \tag{SI.30}$$

be survival just after selection (i.e. one iteration of the breeder's equation) yet before the next time step. Define $\hat{l}_j = \hat{p}_0 \hat{p}_1 \ldots \hat{p}_{j-1}$, $j = 0, 1, \ldots$ with $\hat{l}_0 = 1$. Let $\Delta_{\mathrm{s}}\lambda(t) > 0$ be the increase in growth rate λ with respect to the stationary regime ($\lambda = 1$) that is required so that the Euler-Lotka equation

$$\sum_{j=1}^{\infty} \hat{l}_j(t) m_j (1 + \Delta_{\mathrm{s}}\lambda(t))^{-j} = 1 \tag{SI.31}$$

holds, i.e. $1 + \Delta_{\mathrm{s}}\lambda(t)$ is the asymptotic geometric growth of a population where survival at age $j = 0, 1, \ldots$ is fixed at $\hat{p}_j(t)$. To ensure that the population is again stationary at $t + 1$, so that

$$\sum_{j=1}^{\infty} l_j(t+1) m_j = 1, \tag{SI.32}$$

we have to set $p_j(t+1) = \hat{p}_j(t)/(1 + \Delta_{\mathrm{s}}\lambda(t))$. Survival evolution is then given by the equations

$$p_j(t+1) = \frac{f_{\mathrm{P}}^{-1}\left(f_{\mathrm{P}}(p_j(t)) + \delta \frac{1}{f'_{\mathrm{P}}(p_j(t))} \frac{H_j(t)}{p_j(t)}\right)}{1 + \Delta_{\mathrm{s}}\lambda(t)}, \qquad j = 0, 1, \ldots \tag{SI.33}$$

No mechanism is enforced to ensure that survival cannot exceed 1 during the time evolution of the system. This is deliberately done with the aim of finding out whether sensible choices of f_{P} naturally respect this bound. The problem of whether the dynamics in Eq. (SI.33) are bounded is discussed in Sections 3.1 and 3.7. In what follows, it will be assumed that the bound is respected.

In the numerator of Eq. (SI.33), survival evolves according to Eq. (SI.17) by small increments at the level at which the genetic variance is additive. The denominator imposes ecological equilibrium by scaling survival at all ages by the same factor, see Section 1.4. Permanent demographic stationarity is then obtained, and Eq. (SI.17) can be iterated. Providing survival stays bounded, the dynamics in Eq. (SI.33) ensure its positivity throughout. Since f_{P} is strictly increasing in its argument and the selection gradient on survival is always positive, the numerator in Eq. (SI.33) at t is always greater than $p_j(t)$. The denominator is always positive. Hence, at each time step, p_j is first increased and, then, scaled by a positive quantity thereby keeping p_j positive independently of t.

2 Analysis of fecundity evolution

2.1 Equilibria in fecundity evolution

Here some general properties of equilibria in fecundity evolution are derived. Equilibrium quantities are denoted with an asterisk. Substituting Eq. (SI.26) into Eq. (SI.28), taking a first order Taylor expansion

around δ and using the fact that $\sum_{i=1}^{\infty} l_i m_i = 1$, i.e. the net reproduction rate in the unselected population, one gets

$$1 + \Delta_{\rm s} R(t) \approx \sum_{i=\alpha}^{\infty} l_i \left(m_i(t) + \frac{\delta l_i}{T(t) f'_{\rm M}(m_i(t))^2} \right) = 1 + \frac{\delta}{T(t)} \sum_{i=\alpha}^{\infty} \frac{l_i^2}{f'_{\rm M}(m_i(t))^2}, \tag{SI.34}$$

where the series is assumed to converge because, by weak selection ($0 < \delta \ll 1$), the quantity $\Delta_{\rm s} R(t)$ is assumed small. The series starts at α because fecundities at pre-reproductive ages are zero throughout. Substituting this expression into Eq. (SI.27) and with a first-order Taylor expansion around $\delta = 0$, one gets

$$m_j(t+1) \approx m_j(t) \left[1 + \frac{\delta}{T(t)} \left(\frac{l_j}{m_j(t) f'_{\rm M}(m_j(t))^2} - \sum_{i=1}^{\infty} \frac{l_i^2}{f'_{\rm M}(m_i(t))^2} \right) \right], \qquad j = \alpha, \alpha+1, \dots \tag{SI.35}$$

where the general relationship $(f^{-1}(z))' = 1/f'(f^{-1}(z))$ was used. An equilibrium of Eq. (SI.35) implies that

$$m_{j+1}^* f'_{\rm M}(m_{j+1}^*)^2 = m_j^* f'_{\rm M}(m_j^*)^2 p_j, \quad j = \alpha, \alpha+1, \dots \tag{SI.36}$$

Note that a flat trajectory of fecundity, i.e. $m_\alpha^* = m_{\alpha+1}^* = \dots = m_n^* = \dots$, cannot be an equilibrium, for it would imply $p_j = 1$, $j = \alpha, \alpha+1, \dots$, i.e. the impossibility of death at adult ages.

2.2 Equilibrium selection gradient on fecundity

Rearranging Eq. (SI.36) and using $p_j = l_{j+1}/l_j$ from Eq. (SI.1), one gets

$$\frac{l_{j+1}}{f'_{\rm M}(m_{j+1}^*)} = \frac{l_j}{f'_{\rm M}(m_j^*)} \sqrt{p_j \frac{m_{j+1}^*}{m_j^*}}, \quad j = \alpha, \alpha+1, \dots \tag{SI.37}$$

By Eq. (SI.25b), one has that Eq. (SI.37) expresses a relationship about the equilibrium selection gradient on fecundity at two consecutive ages. The relationship evidences how this gradient has no obvious universal behaviour. However, when at equilibrium there is ageing in fecundity, $m_{j+1}^* < m_j^*$, $j = \alpha, \alpha+1, \dots$, the squared root on the right hand side of Eq. (SI.37) is smaller than 1. Consequently, at any equilibrium with ageing, the selection gradient on fecundity decreases in magnitude with age.

2.3 Sets of functions for fecundity evolution

Aside from the restriction of having a positive first derivative, the function $f_{\rm M}$ requires further specification. Here, two sets of functions are defined to sort out different assumptions on genetic variance additivity that will be shown to lead to qualitatively similar dynamical behaviour. Consider the differential equation

$$(mf'(m)^2)' = f'(m)^2 + 2mf'(m)f''(m) = 0, \quad 0 < m < \infty \tag{SI.38}$$

The general solution of this differential equation on $(0, \infty)$ is of the form $a_1\sqrt{m} + a_2$, where a_1 and a_2 are constants. To ensure a positive first derivative of this solution, we require $a_1 > 0$. But any such solution leads to an unrealistic equilibrium in Eq. (SI.36), as it requires $p_j = 1$, $j = \alpha, \alpha+1, \dots$, i.e. impossibility of death during adult life. This result naturally leads to consider two disjoint sets of functions for the parametrization of the selection gradient on fecundity. These sets are obtained by looking at the differential inequalities corresponding to the differential equation (SI.38).

2.3.1 Definition of $\mathscr{F}_+^m$

The first set is

$$\mathscr{F}_+^m := \{ f_{\rm M} : (mf'_{\rm M}(m)^2)' > 0 \text{ for } 0 < m < \infty \} \tag{SI.39}$$

From Eq. (SI.38), linear and convex (i.e. $f''_{\mathrm{M}}(m) \geq 0$) functions on $(0, \infty)$, e.g. m^q with $q > 1/2$ or $\exp(m)$, form a subset of $\mathscr{F}_+^m$. From the strict monotonicity enclosed in the definition of $\mathscr{F}_+^m$,

$$\text{if } f_{\mathrm{M}} \in \mathscr{F}_+^m, \text{ then } m f'_{\mathrm{M}}(m)^2 \text{ preserves order on } (0, \infty) \tag{SI.40}$$

and $m f'_{\mathrm{M}}(m)^2$ is an invertible function on $(0, \infty)$.

2.3.2 Definition of $\mathscr{F}_-^m$

The second set is

$$\mathscr{F}_-^m := \{f_{\mathrm{M}} : (m f'_{\mathrm{M}}(m)^2)' < 0 \text{ for } 0 < m < \infty\} \tag{SI.41}$$

From Eqs. (SI.23) and (SI.38), if $f_{\mathrm{M}} \in \mathscr{F}_-^m$, then f_{M} is concave (i.e. $f''_{\mathrm{M}}(m) < 0$). Notably, $\ln m$ and m^q with $0 < q < 1/2$ are in $\mathscr{F}_-^m$. From the strict monotonicity enclosed in the definition of $\mathscr{F}_-^m$,

$$\text{if } f_{\mathrm{M}} \in \mathscr{F}_-^m, \text{ then } m f'_{\mathrm{M}}(m)^2 \text{ inverts order on } (0, \infty) \tag{SI.42}$$

and $m f'_{\mathrm{M}}(m)^2$ is an invertible function on $(0, \infty)$.

2.4 Fecundity at equilibrium

Equilibria of the form in Eq. (SI.36), if any exists for the given survival and the chosen f_{M}, can be obtained via a recursive procedure. Using Eq. (SI.1), the equilibrium equations (SI.36) for fecundity can be expressed as

$$m_j^* f'_{\mathrm{M}}(m_j^*)^2 = m_\alpha^* f'_{\mathrm{M}}(m_\alpha^*)^2 \frac{l_j}{l_\alpha}, \qquad j = \alpha, \alpha+1, \ldots \tag{SI.43}$$

Let m_α^* be the unknown z. Taking the inverse function of $z f'_{\mathrm{M}}(z)^2$ on both sides of Eq. (SI.43) allows to express all other equilibrium fecundities solely as functions of z along with survival at some ages. Equilibrium fecundities must also give a net reproduction rate of 1. Therefore, an equilibrium value of m_α is equal to any positive solution of the equation $R(z) = 1$. From this solution and using again the inverse of $z f'_{\mathrm{M}}(z)^2$ on Eq. (SI.43), the corresponding equilibrium values of m_j, $j = \alpha+1, \alpha+2, \ldots$, are obtained.

2.4.1 Ageing in fecundity at equilibrium

Let $f_{\mathrm{M}} \in \mathscr{F}_+^m$. By Eqs. (SI.36) and (SI.40), $m_{j+1}^* < m_j^*$, $j = \alpha, \alpha+1, \ldots$. Therefore, an equilibrium life history, if it exists, shows ageing in fecundity when age-specific genetic variation is additive at the level of $f_{\mathrm{M}}(m_j)$. For some choices of f_{M}, closed forms of the equilibria may be obtained via the strategy explained above. For example, let $f_{\mathrm{M}}(m) = m^q$ with $q > \frac{1}{2}$. Then, Eq. (SI.43) becomes

$$q^2 (m_j^*)^{2q-1} = q^2 (m_\alpha^*)^{2q-1} \frac{l_j}{l_\alpha}, \qquad j = \alpha, \alpha+1, \ldots \tag{SI.44}$$

and taking the inverse function of $m f'_{\mathrm{M}}(m)^2 = q^2 m^{2q-1}$ on both sides

$$m_j^* = m_\alpha^* \left(\frac{l_j}{l_\alpha}\right)^{\frac{1}{2q-1}}, \qquad j = \alpha, \alpha+1, \ldots \tag{SI.45}$$

Substituting this into $R^* = 1$ using Eq. (SI.3),

$$1 = \sum_{j=\alpha}^{\infty} l_j m_j^* = \sum_{j=\alpha}^{\infty} l_j m_\alpha^* \left(\frac{l_j}{l_\alpha}\right)^{\frac{1}{2q-1}} = m_\alpha^* \sum_{j=\alpha}^{\infty} \frac{l_j^{\frac{2q}{2q-1}}}{l_\alpha^{\frac{1}{2q-1}}}, \tag{SI.46}$$

where we start the series at $j = \alpha$ because at pre-reproductive ages $m_j = 0$, $j = 1, 2, \dots, \alpha - 1$. Combining Eqs. (SI.45) and (SI.46),

$$m_j^* = \frac{l_j^{\frac{1}{2q-1}}}{\sum_{i=\alpha}^{\infty} l_i^{\frac{2q}{2q-1}}}, \quad j = \alpha, \alpha+1, \dots, \qquad f_{\mathrm{M}}(m) = m^q \text{ with } q > \frac{1}{2}. \tag{SI.47}$$

A sufficient condition for convergence of the series in the denominator of Eq. (SI.47) is that the survival values, $p_0, p_1, \dots$ which are arbitrary, fixed constants each independently chosen from the interval $(0, 1)$ form a sequence (p_n) the least upper bound of which

$$\sup(p_n) = \breve{p}, \tag{SI.48}$$

is such that

$$\breve{p}^{\frac{2q}{2q-1}} := r < 1. \tag{SI.49}$$

Since $2q/(2q-1) > 0$ for $q > 1/2$, we have that $\breve{p} < 1$ is a sufficient condition for the inequality in Eq. (SI.49) to hold. In this case, we can use Eq. (SI.1) and bound the series contained in Eq. (SI.47) as follows

$$\sum_{i=\alpha}^{\infty} l_i^{\frac{2q}{2q-1}} \leq \sum_{i=\alpha}^{\infty} (\breve{p}^i)^{\frac{2q}{2q-1}} = \sum_{i=\alpha}^{\infty} r^i = \frac{r^\alpha}{1-r} = \frac{\breve{p}^{\frac{2\alpha q}{2q-1}}}{1 - \breve{p}^{\frac{2q}{2q-1}}}. \tag{SI.50}$$

We stress that Eqs. (SI.48)-(SI.49) do not imply the impossibility of negative ageing in survival, see Section 1.2. The sequence of survival may monotonically increase while asymptotically approaching $\breve{p}$ as its limit value.

2.4.2 Negative ageing in fecundity at equilibrium

Let $f_{\mathrm{M}} \in \mathscr{F}_{-}^{m}$. By Eqs. (SI.36) and (SI.42), $m_{j+1}^* > m_j^*$, $j = \alpha, \alpha+1, \dots$. Therefore, an equilibrium life history, if it exists, shows negative ageing for some concave f_{M}. For some choices of $f_{\mathrm{M}} \in \mathscr{F}_{-}^{m}$, closed forms of the equilibria may be obtained via the strategy explained above. For example, the same expression as in Eq. (SI.47) is obtained for $f_{\mathrm{M}}(m) = m^q$ with $0 < q < \frac{1}{2}$, which is a function in $\mathscr{F}_{-}^{m}$. Hence,

$$m_j^* = \frac{l_j^{\frac{1}{2q-1}}}{\sum_{i=\alpha}^{\infty} l_i^{\frac{2q}{2q-1}}}, \quad j = \alpha, \alpha+1, \dots, \qquad f_{\mathrm{M}}(m) = m^q \text{ with } 0 < q < \frac{1}{2}. \tag{SI.51}$$

However, we have $l_i = p_0 p_1 \dots p_{i-1} < 1$ for each term in the series in the denominator of Eq. (SI.51). This is because $p_0, p_1, \dots$ are arbitrary, fixed constants each independently chosen from the interval $(0, 1)$. Since $2q/(2q-1) < 0$ for $0 < q < 1/2$, each term in the series in the denominator of Eq. (SI.51) exceeds 1. Therefore, the series diverges and an equilibrium fecundity schedule is not found for $f_{\mathrm{M}}(m) = m^q$ with $0 < q < \frac{1}{2}$.

The attempt of deriving expressions for equilibria when $f_{\mathrm{M}} \in \mathscr{F}_{-}^{m}$ reveals that also in other cases no equilibrium of the form $m_{j+1}^* > m_j^*$, $j = \alpha, \alpha+1, \dots$ is possible. An example is when $f_{\mathrm{M}} = \ln(m)$. Suppose, towards a contradiction, that an equilibrium of the form $m_{j+1}^* > m_j^*$, $j = \alpha, \alpha+1, \dots$ exists when $f_{\mathrm{M}} = \ln(m)$. Then, Eq. (SI.43) becomes

$$\frac{1}{m_j^*} = \frac{1}{m_\alpha^*} \frac{l_j}{l_\alpha}, \qquad j = \alpha, \alpha+1, \dots \tag{SI.52}$$

and taking the inverse function of $m f_{\mathrm{M}}'(m)^2 = m^{-1}$ on both sides

$$m_j^* = m_\alpha^* \frac{l_\alpha}{l_j}, \qquad j = \alpha, \alpha+1, \dots \tag{SI.53}$$

Substituting this into $R^* = 1$ using Eq. (SI.3),

$$1 = \sum_{j=\alpha}^{\infty} l_j m_j^* = \sum_{j=\alpha}^{\infty} l_\alpha m_\alpha^* = l_\alpha m_\alpha^* \sum_{j=\alpha}^{\infty} 1. \tag{SI.54}$$

where the last series on the right diverges. Therefore, we have obtained a contradiction and no equilibrium of the form $m_{j+1}^* > m_j^*$, $j = \alpha, \alpha+1, \ldots$ exists in our model when $f_\mathrm{M} = \ln(m)$. Equilibria when $f_\mathrm{M}(m) = m^q$ with $0 < q < \frac{1}{2}$ or $f_\mathrm{M} = \ln(m)$ exist, if a maximum age ω is imposed, i.e. $p_\omega = 0$.

2.4.3 Uniqueness

Here, it is shown that if the dynamics in Eq. (SI.35) have an equilibrium of the form in Eq. (SI.36), then this equilibrium is unique. As seen above, an equilibrium point (m_α^*, m_j^*), $j = \alpha, \alpha+1, \ldots$, in the m_α-m_j plane satisfies Eq. (SI.43). Therefore, any such point is located on the curve defined by the equation

$$\frac{m_j f'_\mathrm{M}(m_j)^2}{m_\alpha f'_\mathrm{M}(m_\alpha)^2} = \frac{l_j}{l_\alpha}, \quad j = \alpha, \alpha+1, \ldots \tag{SI.55}$$

in the m_α-m_j plane. Implicit differentiation on Eq. (SI.55) can be used to find the slope of this curve,

$$\begin{aligned} \frac{\mathrm{d}m_j}{\mathrm{d}m_\alpha} &= -\left[\frac{\partial}{\partial m_\alpha}\left(\frac{m_j f'_\mathrm{M}(m_j)^2}{m_\alpha f'_\mathrm{M}(m_\alpha)^2}\right)\right]\left[\frac{\partial}{\partial m_j}\left(\frac{m_j f'_\mathrm{M}(m_j)^2}{m_\alpha f'_\mathrm{M}(m_\alpha)^2}\right)\right]^{-1} \\ &= \frac{m_j f'_\mathrm{M}(m_j)^2}{m_\alpha f'_\mathrm{M}(m_\alpha)^2}\left(\frac{\partial(m_\alpha f'_\mathrm{M}(m_\alpha)^2)}{\partial m_\alpha}\right)\left(\frac{\partial(m_j f'_\mathrm{M}(m_j)^2)}{\partial m_j}\right)^{-1} > 0, \quad j = \alpha, \alpha+1, \ldots \end{aligned} \tag{SI.56}$$

The product on the right hand side of the second line of this expression is always positive, because the first factor is always positive, while the last two factors always have the same sign. In particular, they are both positive when $f_\mathrm{M} \in \mathscr{F}_+^m$, while they are both negative when $f_\mathrm{M} \in \mathscr{F}_-^m$. Therefore, Eq. (SI.55) defines a curve along which m_j is strictly increasing in m_α. Assume then that an equilibrium of the form in Eq. (SI.36) exists. For each $j = \alpha, \alpha+1, \ldots$, there is a point (m_α^*, m_j^*) on the curve defined by Eq. (SI.55). Recall that, because of the assumption of demographic stationarity, $1 = R^* = \sum_{i=\alpha}^{\infty} l_i m_i^*$, where we omit terms with index smaller than α because fecundity is zero at pre-reproductive ages.

Suppose now, towards a contradiction, that some other equilibrium of the form in Eq. (SI.36) exists. We indicate this other putative equilibrium with two asterisks. Then, for at least one j, there is a point $(m_\alpha^{**}, m_j^{**})$ different from (m_α^*, m_j^*) yet located on the same curve given by Eq. (SI.55). As the two points are distinct, one can express $(m_\alpha^{**}, m_j^{**}) = (m_\alpha^* + \varepsilon_\alpha, m_j^* + \varepsilon_j)$ for ε_α and ε_j not both zero. But $\varepsilon_\alpha > 0$ if and only if $\varepsilon_j > 0$ and $\varepsilon_\alpha < 0$ if and only if $\varepsilon_j < 0$ because of Eq. (SI.56), i.e. while moving along the curve in Eq. (SI.20), any increase (decrease) in m_α corresponds to an increase (decrease) in m_j. Therefore, either $m_j^{**} < m_j^*$, $j = \alpha, \alpha+1, \ldots$, or $m_j^{**} > m_j^*$, $j = \alpha, \alpha+1, \ldots$. Either way,

$$R^{**} = \sum_{i=\alpha}^{\infty} l_i m_i^{**} \neq \sum_{i=\alpha}^{\infty} l_i m_i^* = R^* = 1 \tag{SI.57}$$

But this contradicts the assumption of demographic stationarity. Therefore, if there is an equilibrium of the form in Eq. (SI.36), then this equilibrium is unique.

2.5 Dynamical properties of fecundity evolution

2.5.1 Coordinate transformation

Some key dynamical properties of fecundity evolution are better understood with a coordinate transformation. The original system in Eq. (SI.27) is in the variables $m_\alpha, m_{\alpha+1}, \ldots$, i.e. a sequence (m_n), which

must be such that $R = 1$ at all times $t = 0, 1, \dots$ for the chosen survival probabilities. Consider then the transformed system in the variables

$$y_j := \frac{m_{j+1}}{m_j}, \quad j = \alpha, \alpha+1, \dots \tag{SI.58}$$

which form another sequence $(y_n) = y_\alpha, y_{\alpha+1}, \dots$ that is well defined when $m_j > 0$, $j = \alpha, \alpha+1, \dots$. In this regard, recall that the positivity of fecundity is guaranteed by the dynamics in Eq. (SI.27). The transformation in Eq. (SI.58) can be shown invertible. Take the sequence (m_n), which satisfies $R = 1$ for the chosen survival probabilities. Let the term m_α of this sequence to be the unknown $u > 0$. Note that

$$m_k = u \prod_{i=\alpha}^{k-1} y_i, \quad k = \alpha, \alpha+1, \dots \tag{SI.59}$$

Write then R in Eq. (SI.3) using Eq. (SI.59) and the given survival values,

$$R(u) = \sum_{i=\alpha}^{\infty} u \left(l_i \prod_{j=\alpha}^{i-1} y_j \right), \tag{SI.60}$$

where the series converges to 1 for some value of u in $(0, 1)$. Since $R'(u) > 0$ and $R(0) = 0$, there is a unique value $\hat{u}$ of u in $(0, 1)$ such that $R(\hat{u}) = 1$. Hence, to the sequence $y_0, y_1, \dots$ there corresponds corresponds a unique value $m_\alpha = \hat{u}$ for the first term of the original sequence (m_n). Via Eq. (SI.59), the whole original sequence (m_n) of fecundity is uniquely determined and, therefore, the transformation is invertible. In the following analysis of the transformed system, the quantities m_j, $j = 0, 1, \dots$, should be seen as functions of the sequence (y_n), as seen above.

2.5.2 Dynamics of the transformed system

Taking ratios of the equations of the system in Eq. (SI.27) and with a first order Taylor expansion around $\delta = 0$, the dynamics of the transformed system are given by difference equations of the form

$$\begin{aligned} \Delta y_j(t) &:= y_j(t+1) - y_j(t) \\ &= \frac{f_{\mathrm{M}}^{-1}\left(f_{\mathrm{M}}(m_{j+1}(t)) + \delta \frac{l_{j+1}}{T(t) f'_{\mathrm{M}}(m_{j+1}(t))}\right)}{f_{\mathrm{M}}^{-1}\left(f_{\mathrm{M}}(m_j(t)) + \delta \frac{l_j}{T(t) f'_{\mathrm{M}}(m_j(t))}\right)} - y_j(t) \\ &\approx \delta c_j^m(t) \left[p_j - \xi_j(t)\right], \qquad\qquad j = \alpha, \alpha+1, \dots \end{aligned} \tag{SI.61}$$

where

$$c_j^m(t) = \frac{l_j}{T(t) m_j(t) f'_{\mathrm{M}}(m_{j+1}(t))^2} > 0, \tag{SI.62}$$

and

$$\xi_j(t) = \frac{m_{j+1}(t) f'_{\mathrm{M}}(m_{j+1}(t))^2}{m_j(t) f'_{\mathrm{M}}(m_j(t))^2}. \tag{SI.63}$$

Here, $c_j^m(t)$ always is greater than zero because it is a product of positive quantities. Consequently, the sign of $\Delta y_j(t)$ only depends on the difference in brackets in Eq. (SI.61). Supposing that $y_j(t) > 0$, $j = \alpha, \alpha+1, \dots$, for all t, the system can always be made correspond to the original system in Eq. (SI.27), as shown in the previous section. Working with Eq. (SI.61) seems complicated because of the laborious evaluation of $m_\alpha, m_{\alpha+1}, \dots$, which are here each a function of the sequence (y_n). However, the transformed system can still help us to gain theoretical understanding of the original system as the next sections will show.

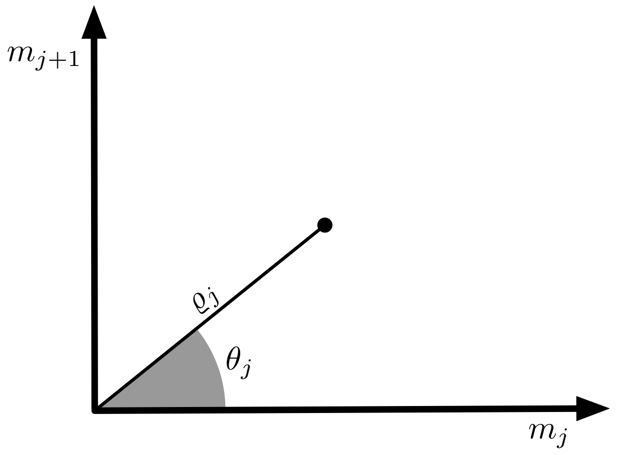

Figure SI.1: **Age-specific fecundity at two successive ages in polar coordinates**

2.5.3 Basic properties of the transformed system

Recalling that $mf'_{\mathrm{M}}(m)^2 > 0$, some basic properties connecting y_j and ξ_j, $j = \alpha, \alpha+1, \ldots$, are readily obtained. From Eq. (SI.40),

$$y_j > 1 \quad \text{if and only if} \quad \xi_j > 1, \qquad f_{\mathrm{M}} \in \mathscr{F}_+^m, \tag{SI.64a}$$

$$y_j < 1 \quad \text{if and only if} \quad \xi_j < 1, \qquad f_{\mathrm{M}} \in \mathscr{F}_+^m. \tag{SI.64b}$$

From Eq. (SI.42),

$$y_j > 1 \quad \text{if and only if} \quad \xi_j < 1, \qquad f_{\mathrm{M}} \in \mathscr{F}_-^m, \tag{SI.65a}$$

$$y_j < 1 \quad \text{if and only if} \quad \xi_j > 1, \qquad f_{\mathrm{M}} \in \mathscr{F}_-^m, \tag{SI.65b}$$

and from Eqs. (SI.40) and (SI.42),

$$y_j = 1 \quad \text{if and only if} \quad \xi_j = 1, \qquad f_{\mathrm{M}} \in \mathscr{F}_-^m \text{ or } f_{\mathrm{M}} \in \mathscr{F}_+^m. \tag{SI.66}$$

Further properties are derived by noting that any given positive value of y_j corresponds to the slope of a line through the origin in the m_j-m_{j+1} plane. This line is the set of all ordered pairs (m_j, m_{j+1}) corresponding to the same y_j value. Using polar coordinates, let θ_j be the angle between the (horizonal) m_j-axis and the line going through the origin and the point (m_j, m_{j+1}), which lies in a circle of radius ϱ_j, see Fig. SI.1.

Let us then switch to polar coordinates, $\xi_j(\theta_j, \varrho_j)$, where $\varrho_j = \sqrt{m_j^2 + m_{j+1}^2}$ and $\theta_j = \arctan\left(\frac{m_{j+1}}{m_j}\right) = \arctan y_j$, such that $m_{j+1} = \varrho_j \sin(\theta_j)$ and $m_j = \varrho_j \cos(\theta_j)$. Differentiating with respect to the angle,

$$\begin{aligned}\frac{\partial \xi_j}{\partial \theta_j} &= \frac{\partial m_{j+1}}{\partial \theta_j}\frac{\partial \xi_j}{\partial m_{j+1}} + \frac{\partial m_j}{\partial \theta_j}\frac{\partial \xi_j}{\partial m_j} \\ &= m_j \frac{\partial \xi_j}{\partial m_{j+1}} - m_{j+1}\frac{\partial \xi_j}{\partial m_j} \\ &= \frac{1}{f'_{\mathrm{M}}(m_j)^2}\left[\frac{\partial(m_{j+1} f'_{\mathrm{M}}(m_{j+1})^2)}{\partial m_{j+1}} + y_j \xi_j \left(\frac{\partial(m_j f'_{\mathrm{M}}(m_j)^2)}{\partial m_j}\right)\right].\end{aligned} \tag{SI.67}$$

From Eqs. (SI.39), (SI.41) and (SI.67), one has that

$$\begin{aligned}\frac{\partial \xi_j}{\partial \theta_j} &> 0, \qquad f_{\mathrm{M}} \in \mathscr{F}_+^m, \\ \frac{\partial \xi_j}{\partial \theta_j} &< 0, \qquad f_{\mathrm{M}} \in \mathscr{F}_-^m.\end{aligned} \tag{SI.68}$$

Differentiating then with respect to the radius, we obtain

$$
\begin{aligned}
\frac{\partial \xi_j}{\partial \varrho_j} &= \frac{\partial m_{j+1}}{\partial \varrho_j}\frac{\partial \xi_j}{\partial m_{j+1}} + \frac{\partial m_j}{\partial \varrho_j}\frac{\partial \xi_j}{\partial m_j} \\
&= \frac{1}{\varrho_j}\left(m_{j+1}\frac{\partial \xi_j}{\partial m_{j+1}} + m_j\frac{\partial \xi_j}{\partial m_j}\right) \\
&= \frac{\xi_j}{\sqrt{m_j^2+m_{j+1}^2}}\left[\frac{1}{f'_{\mathrm{M}}(m_{j+1})^2}\frac{\partial(m_{j+1}f'_{\mathrm{M}}(m_{j+1})^2)}{\partial m_{j+1}} - \frac{1}{f'_{\mathrm{M}}(m_j)^2}\frac{\partial(m_j f'_{\mathrm{M}}(m_j)^2)}{\partial m_j}\right].
\end{aligned}
\tag{SI.69}
$$

The quantity between brackets in (SI.69) vanishes for $m_j = m_{j+1}$ corresponding to $\theta_j = \frac{\pi}{4}$,

$$
\left.\frac{\partial \xi_j}{\partial \varrho_j}\right|_{\theta_j=\frac{\pi}{4}} = 0. \tag{SI.70}
$$

The problem of establishing dynamical properties of the transformed system can now be tackled.

2.5.4 Set stability of fecundity

The aim here is to characterize regions of the state space of the transformed system that attract or repel the dynamics of the sequence $(y_n) = y_\alpha, y_{\alpha+1}, \ldots$. Define the sets

$$
\begin{aligned}
U^m &:= \{(y_n) : 0 < y_j, && j = \alpha, \alpha+1, \ldots\} \\
A^m_{\leq} &:= \{(y_n) : 0 < y_j \leq 1, && j = \alpha, \alpha+1, \ldots\} \\
A^m_{<} &:= \{(y_n) : 0 < y_j < 1, && j = \alpha, \alpha+1, \ldots\} \\
A^m_{>} &:= \{(y_n) : y_j > 1, && j = \alpha, \alpha+1, \ldots\}
\end{aligned}
\tag{SI.71}
$$

so that U^m is the entire state space where the dynamics of (y_n) unfold. The possibility that $y_j = 0$ for some $j = \alpha, \alpha+1, \ldots$ is excluded. This is because fecundity is assumed initially positive and the dynamics in Eq. (SI.27) should guarantee its strict positivity throughout. In the transformed system, this should preserve the positivity of y_j, $j = \alpha, \alpha+1, \ldots$ throughout.

Any complement of a subset of U^m should be meant as a complement relative to U^m excluding the zero boundary. The set $A^m_{\leq}$ is the set of all life histories with fecundity that either stays constant or decreases with age. By the definitions in Section 1.2, $A^m_{<}$ is the set of all life histories with ageing in fecundity, while $A^m_{>}$ is the set of all life histories with negative ageing in fecundity. Note that $A^m_{<}$ is the interior of $A^m_{\leq}$. The dynamical behaviour induced by the two sets of functions defined in Section 2.3 is analysed separately.

Case 1: $f_{\mathrm{M}} \in \mathscr{F}^m_+$.

As shown in Section 2.4.1, at equilibrium, $m^*_{j+1} < m^*_j$, $j = \alpha, \alpha+1, \ldots$. Therefore, $y^*_j < 1$, $j = \alpha, \alpha+1, \ldots$ and $(y^*_n) \in A^m_{<} \subset A^m_{\leq}$. Here, three properties of $A^m_{\leq}$ are shown under the dynamics in Eq. (SI.61) assuming sufficiently weak selection:

(i) $A^m_{\leq}$ is an invariant set: if $(y_n(t)) \in A^m_{\leq}$, then $(y_n(t+1)) \in A^m_{\leq}$. To show this, observe that all y_j change by very small amounts at each time step because of weak selection. Hence, the passage from $y_j(t) \leq 1$ to $y_j(t+1) > 1$ for some $j = \alpha, \alpha+1, \ldots$ will happen in some small neighbourhood at the boundary between $A^m_{\leq}$ and its complement. In this neighbourhood, $y_j \approx 1$. Therefore, $m_j \approx m_{j+1}$ and $\theta_j \approx \frac{\pi}{4}$ in this neighbourhood.

By Eq. (SI.70), the derivative $\frac{\partial \xi_j}{\partial \varrho_j}$ tends to vanish in this neighbourhood. Since $\theta_j = \arctan(y_j)$ only is a function of y_j we can write the total differential

$$
\mathrm{d}\xi_j \approx \frac{\partial \xi_j}{\partial y_j}\Delta y_j = \frac{\partial \theta_j}{\partial y_j}\frac{\partial \xi_j}{\partial \theta_j}\Delta y_j = \frac{1}{1+y_j^2}\frac{\partial \xi_j}{\partial \theta_j}\Delta y_j, \tag{SI.72}
$$

only in terms of the change in y_j and the sensitivity of ξ_j to it, because the y_i with $i \neq j$ have an influence on ξ_j only via ϱ_j. This differential is meant to be valid when $y_j \approx 1$. We also note that, in this differential, the quantity $\frac{\partial \xi_j}{\partial \theta_j}$ is positive by Eq. (SI.68).

Suppose now, towards a contradiction, that $(y_n(t)) \in A^m_{\leq}$ and $(y_n(t+1)) \notin A^m_{\leq}$. Then, by Eq. (SI.71), $y_j(t) \leq 1$, for all $j = \alpha, \alpha+1, \dots$, while $y_j(t+1) > 1$ for at least some $j = \alpha, \alpha+1, \dots$. Let $K \subseteq \{\alpha, \alpha+1, \dots\}$ be the index set such that $j \in K$ if and only if $y_j(t+1) > 1$. Then, $y_j(t+1) > 1 \geq y_j(t)$ for $j \in K$. This implies, by Eq. (SI.61), that $p_j - \xi_j(t) > 0$ for $j \in K$ and, by Eq. (SI.64), that $\xi_j(t+1) > 1$ for $j \in K$. But $p_j < 1$ for all j. Hence, using the total differential $\mathrm{d}\xi_j(t)$ in Eq. (SI.72) as a substitute for $\Delta\xi_j(t) = \xi_j(t+1) - \xi_j(t)$,

$$p_j < \xi_j(t+1) \approx \xi_j(t) + \frac{\Delta y_j(t)}{1 + y_j^2(t)} \frac{\partial \xi_j}{\partial \theta_j}(t), \quad j \in K. \tag{SI.73}$$

Subtracting $\xi_j(t)$ from both sides of this inequality and then dividing by the positive quantity $p_j - \xi_j(t)$ and using Eq. (SI.61),

$$1 < \delta \frac{c_j^m(t)}{1 + y_j^2(t)} \frac{\partial \xi_j}{\partial \theta_j}(t), \quad j \in K. \tag{SI.74}$$

But this contradicts the assumption of weak selection (i.e. $0 < \delta \ll 1$). Therefore, it is not possible that $(y_n(t)) \in A^m_{\leq}$ and $(y_n(t+1)) \notin A^m_{\leq}$. Equivalently, if $(y_n(t)) \in A^m_{\leq}$, then $(y_n(t+1)) \in A^m_{\leq}$.

(ii) $A^m_{\leq}$ pushes trajectories into its interior: if $(y_n(t)) \in A^m_{\leq}$, then $(y_n(t+1)) \in A^m_{<}$. To show this, suppose $(y_n(t)) \in A^m_{\leq}$. If $y_j(t) = 1$ for some $j = \alpha, \alpha+1, \dots$, then, by Eq. (SI.66), $\xi_j(t) = 1$. But $p_j < 1$ for all j. Hence, $p_j - \xi_j(t) < 0$ and, by Eq. (SI.61), $y_j(t+1) < y_j(t) = 1$. If $y_j(t) < 1$ for some $j = \alpha, \alpha+1, \dots$, then the supposition that $\xi_j(t+1) = 1$ along with the assumption of weak selection leads to the same contradiction as in Eq. (SI.74) via an argument analogous to that used to prove invariance of $A^m_{\leq}$. Thus, if $y_j(t) < 1$, then $y_j(t+1) < 1$.

Therefore, if $y_j(t) \leq 1$, then $y_j(t+1) < 1$ for all $j = \alpha, \alpha+1, \dots$.

(iii) $A^m_{\leq}$ is a stable attracting set. Trajectories from outside of $A^m_{\leq}$ end up in it and stay there. To show this, we combine Eqs. (SI.7) and (SI.10) with the fact that the sequence $1, 1, \dots$ is the boundary of $A^m_{\leq}$ to get the distance of the sequence (y_n) from the set $A^m_{\leq}$ as

$$d_\alpha((y_n), A^m_{\leq}) = \sum_{i=\alpha}^{\infty} \frac{\Theta(y_i - 1)}{2^i} \frac{|y_i - 1|}{1 + |y_i - 1|} \tag{SI.75}$$

where Θ is the unit step function, i.e. $\Theta(z) = 1$ for $z \geq 0$ and $\Theta(z) = 0$ for $z < 0$. The unit step function takes care of the fact that if any term of (y_n) is smaller than 1, then the contribution of the corresponding term in the series in Eq. (SI.75) must be zero, because there is some sequence in $A^m_{\leq}$ that has a term that has both the same index and the same value. Suppose $(y_n(t)) \notin A^m_{\leq}$ so that $d_\alpha((y_n(t)), A^m_{\leq}) > 0$. Then, $y_j(t) > 1$ for some $j = \alpha, \alpha+1, \dots$. Let $K \subseteq \{\alpha, \alpha+1, \dots\}$ be the index set such that $j \in K$ if and only if $y_j(t) > 1$, i.e. K contains the indexes of the positive terms in the series given by $d_\alpha((y_n(t)), A^m_{\leq})$, while all other terms are equal to zero. Let us first consider those terms that are equal to zero in this series. By invariance of $A^m_{\leq}$, if $y_j(t) \leq 1$ for $j \notin K$, then $y_j(t+1) \leq 1$ for $j \notin K$. Thus, for any term that is equal to zero in the series given by $d_\alpha((y_n(t)), A^m_{\leq})$, the corresponding term in the series given by $d_\alpha((y_n(t+1)), A^m_{\leq})$ is still equal to zero. Consider then terms that are greater than zero in the series given by $d_\alpha((y_n(t)), A^m_{\leq})$. By Eq. (SI.64),

$\xi_j(t) > 1$ for $j \in K$. But $p_j < 1$, $j = 0, 1, \ldots$. By Eq. (SI.61), $y_j(t+1) < y_j(t)$. Recall that $y_j(t) - 1 > 0$ for $j \in K$. If $y_j(t+1) - 1 \leq 0$, then

$$\begin{aligned} \frac{\Theta(y_j(t+1)-1)|y_j(t+1)-1|}{1+|y_j(t+1)-1|} &= 0 \\ &< \frac{y_j(t)-1}{1+y_j(t)-1} \\ &= \frac{\Theta(y_j(t)-1)|y_j(t)-1|}{1+|y_j(t)-1|}, \qquad j \in K. \end{aligned} \tag{SI.76}$$

If $y_j(t+1) - 1 > 0$, then, using the fact that $z/(1+z)$ is strictly increasing on $(-1/2, \infty)$ and, therefore, preserves order on this interval,

$$\begin{aligned} \frac{\Theta(y_j(t+1)-1)|y_j(t+1)-1|}{1+|y_j(t+1)-1|} &= \frac{y_j(t+1)-1}{1+y_j(t+1)-1} \\ &< \frac{y_j(t)-1}{1+y_j(t)-1} \\ &= \frac{\Theta(y_j(t)-1)|y_j(t)-1|}{1+|y_j(t)-1|}, \qquad j \in K. \end{aligned} \tag{SI.77}$$

Either way, all positive terms in the series given by $d_\alpha((y_n(t)), A^m_{\leq})$ are larger than the corresponding terms in the series given by $d_\alpha((y_n(t+1)), A^m_{\leq})$. Hence, $d_\alpha((y_n(t+1)), A^m_{\leq}) < d_\alpha((y_n(t)), A^m_{\leq})$. By monotone convergence, we then have that

$$\lim_{t\to\infty} d_\alpha((y_n(t)), A^m_{\leq}) = 0 \tag{SI.78}$$

for any $(y_n(t)) \in U^m$.

Hence, when $f_{\mathrm{M}} \in \mathscr{F}^m_+$, the set $A^m_{\leq}$ fulfils the properties [16] to be a globally stable attracting set of the dynamics of (y_n). In virtue of property (ii), all trajectories lead to $A^m_{<}$ so that the distance between this set and (y_n) also goes to zero. This distance is also measured via Eq. (SI.75) because the distance between an open set and a point on its boundary is zero. Eventually, ageing in fecundity evolves from any initial life history.

Case 2: $f_{\mathrm{M}} \in \mathscr{F}^m_-$.
As shown in Section 2.4.2, at an equilibrium (y^*_n) of the form in Eq. (SI.36) we have $y^*_j > 1$, $j = \alpha, \alpha+1, \ldots$ so that $(y^*_n) \in A^m_{>}$. Here it is shown that the set $A^m_{>}$, which contains any such equilibrium when $f_{\mathrm{M}} \in \mathscr{F}^m_-$, is a repelling set under the dynamics in Eq. (SI.61). Combining Eqs. (SI.7) and (SI.10), the distance between (y_n) and $A^m_{>}$ is

$$d_\alpha((y_n), A^m_{>}) = \sum_{i=\alpha}^{\infty} \frac{\Theta(-(y_i-1))}{2^i} \frac{|y_i-1|}{1+|y_i-1|}, \tag{SI.79}$$

where the unit step function Θ takes care of the fact that if any term of (y_n) is greater than 1, then the contribution of the corresponding term in the series in Eq. (SI.79) must be zero, because there is some sequence in $A^m_{>}$ that has a term that has both the same index and the same value. We also note that if $y_j = 1$ for $j = \alpha, \alpha+1, \ldots$, then the distance in Eq. (SI.79) is zero, as it should be, since the distance between a point on the boundary of an open set and this set is zero. Suppose $(y_n(t)) \notin A^m_{>}$. Then, by Eq. (SI.71), $y_j(t) \leq 1$ for some $j = \alpha, \alpha+1, \ldots$, and

$$d_\alpha((y_n(t)), A^m_{>}) > 0. \tag{SI.80}$$

Let $K \subseteq \{\alpha, \alpha+1, \dots\}$ be the index set such that $j \in K$ if and only if $y_j(t) \leq 1$. By Eqs. (SI.65)-(SI.66), $\xi_j(t) \geq 1$ for $j \in K$. But $p_j < 1$, $j = \alpha, \alpha+1, \dots$. Thus, $p_j - \xi_j(t) < 0$ and, by Eq. (SI.61), $y_j(t+1) < y_j(t)$ for $j \in K$. Therefore, $y_j(t+1) < y_j(t) \leq 1$ for $j \in K$. Using the fact that $z/(1+z)$ is strictly increasing on $(-1/2, \infty)$ and, therefore, preserves order on this interval,

$$\begin{aligned} \frac{\Theta(-(y_j(t+1)-1))|y_j(t+1)-1|}{1+|y_j(t+1)-1|} &= \frac{|y_j(t+1)-1|}{1+|y_j(t+1)-1|} \\ &= \frac{1-y_j(t+1)}{1+1-y_j(t+1)} \\ &> \frac{1-y_j(t)}{1+1-y_j(t)} \\ &= \frac{\Theta(-(y_j(t)-1))|y_j(t)-1|}{1+|y_j(t)-1|}, \qquad j \in K. \end{aligned} \tag{SI.81}$$

Therefore, all positive terms in the series given by $d_\alpha((y_n(t+1)), A^m_>)$ are larger than the corresponding terms in the series given by $d_\alpha((y_n(t)), A^m_>)$. As for the zero terms in the series given by $d_\alpha((y_n(t)), A^m_>)$, the corresponding terms in the series given by $d_\alpha((y_n(t+1)), A^m_>)$ are either zero or positive. Either way, we have that

$$d_\alpha((y_n(t+1)), A^m_>) > d_\alpha((y_n(t)), A^m_>) > 0. \tag{SI.82}$$

Hence, if $(y_n(t)) \notin A^m_>$, then $(y_n(t+1)) \notin A^m_>$ and the distance between $(y_n(t))$ and the set $A^m_>$, where an equilibrium (if any exists) is found, keeps increasing with time. The set of life histories with negative ageing in fecundity $A^m_>$ is repelling. Negative ageing in fecundity can not evolve from an initial life history without negative ageing.

2.5.5 Stability of ageing in fecundity — Power functions

Using the Lyapunov method, the stability at an equilibrium of the transformed system is here explored when $f_{\mathrm{M}}(m) = m^q$ with $q > 1/2$. From Eqs. (SI.58) and (SI.63), we can write

$$\xi_j = y_j^{2q-1}, \qquad \text{with } f_{\mathrm{M}}(m) = m^q \tag{SI.83}$$

which shows that, in this case, ξ_j only depends on y_j and $\xi'_j(y_j) = (2q-1)y_j^{2(q-1)}$. Since for $q > 1/2$, we have $m^q \in \mathscr{F}^m_+$, if there is an equilibrium of the form in Eq. (SI.36), this is in $A^m_<$. Define

$$V^m = \sum_{i=\alpha}^{\infty} \frac{1}{2^i} \frac{(p_i - y_i^{2q-1})^2}{1 + (p_i - y_i^{2q-1})^2} \tag{SI.84}$$

where $(p_n) = p_0, p_1, \dots$ is the constant sequence of survival and $(y_n^{2q-1}) = y_\alpha^{2q-1}, y_{\alpha+1}^{2q-1} \dots$ is a sequence that evolves with time. The quantity $p_j - y_j^{2q-1}$, $j = \alpha, \alpha+1, \dots$, in Eq. (SI.84) corresponds to the bracketed quantity in Eq. (SI.61) when $f_{\mathrm{M}}(m) = m^q$. The series on the left hand side of Eq. (SI.84) is convergent in virtue of Eq. (SI.8) and is always positive except at equilibrium where it vanishes, as the dynamics in Eq. (SI.61) show. Therefore, V^m is a candidate Lyapunov function.

Suppose $(y_n(t))$ is not at equilibrium at t so that $V^m(t) > 0$. Let $K \subseteq \{\alpha, \alpha+1, \dots\}$ be the index set such that $j \in K$ is and only if $(p_j - y_j^{2q-1}(t))^2 > 0$. Note that if $(p_j - y_j^{2q-1}(t))^2 = 0$ for some $j \notin K$, then, by Eq. (SI.61), $y_j(t+1) = y_j(t)$ and $y_j^{2q-1}(t+1) = y_j^{2q-1}(t)$ and, therefore, $(p_i - y_i^{2q-1}(t+1))^2 = 0$. Thus, for any term that is equal to zero in the series given by $V^m(t)$, the corresponding term in the series given by $V^m(t+1)$ is still equal to zero. Consider then terms in the series given by $V^m(t)$ with index in K. Neglecting terms of order δ^2 and using Eq. (SI.61) to approximate both $y_j^{2q-1}(t+1)$ and $y_j^{2(2q-1)}(t+1)$

with first-order Taylor expansions around $\delta = 0$ and to get an expression for $\Delta y_j(t) := y_j(t+1) - y_j(t)$, we have that

$$\begin{aligned}(p_j - y_j^{2q-1}(t+1))^2 - (p_j - y_j^{2q-1}(t))^2 \approx & \quad -2p_j\Delta y_j(t)(2q-1)y_j^{2(q-1)}(t) \\ & \quad + 2y_j^{2q-1}(t)\Delta y_j(t)(2q-1)y_j^{2(q-1)}(t) \\ = & \quad -2(2q-1)\delta c_j^m(t)(p_j - y_j^{2q-1}(t))^2 y_j^{2(q-1)}(t) \\ < 0, & \quad j \in K.\end{aligned} \tag{SI.85}$$

The function $z/(1+z)$ is strictly increasing on $(-1/2, \infty)$ and preserves order on this interval. Therefore,

$$\text{if } V^m(t) > 0, \text{ then } V^m(t+1) - V^m(t) < 0, \quad f_{\mathrm{M}}(m) = m^q \text{ with } q > 1/2, \tag{SI.86}$$

and V^m is a strict Lyapunov function: there is some $(y_n^*) \in A_<^m$ that is a unique, globally stable equilibrium of the transformed system when $f_{\mathrm{M}}(m) = m^q$ with $q > 1/2$. Transforming back to the original system in (m_n), there is a unique globally stable life history with ageing in fecundity when $f_{\mathrm{M}}(m) = m^q$ with $q > 1/2$.

2.5.6 Stability of ageing in fecundity — Exponential functions

Stability at equilibrium for the original (non-transformed) system is here explored for functions f_{M} of the exponential family. In particular, we focus on solutions of the differential equation

$$\frac{f''_{\mathrm{M}}(m)}{f'_{\mathrm{M}}(m)} = c > 0 \tag{SI.87}$$

where c is a constant. This differential equation has the solution

$$f_{\mathrm{M}}(m) = a_1 \frac{\exp(cm)}{c} + a_2 \tag{SI.88}$$

where $a_1 > 1$ and a_2 are constants. This solution is in $\mathscr{F}_+^m$, since $f''_{\mathrm{M}}(m) > 0$.

Using Eq. (SI.35), define the map

$$F_j((m_n(t))) = m_j(t+1) = m_j(t) + \frac{\delta}{T(t)}\left(\frac{l_j}{f'_{\mathrm{M}}(m_j(t))^2} - m_j(t)\sum_{i=\alpha}^{\infty}\frac{l_i^2}{f'_{\mathrm{M}}(m_i(t))^2}\right), \quad j = \alpha, \alpha+1, \ldots, \tag{SI.89}$$

which takes as argument the sequence (m_n) of fecundity at t and returns the term m_j, $j = \alpha, \alpha+1, \ldots$ of the sequence (m_n) of fecundity at $t+1$. Suppose there exists an equilibrium sequence (m_n^*) of fecundity for $f_{\mathrm{M}}(m)$ of the form in Eq. (SI.88). We then differentiate the map in Eq. (SI.89),

$$\left.\frac{\partial F_j}{\partial m_i}\right|_{(m_n)=(m_n^*)} = \begin{cases} 1 + \dfrac{\delta}{T^*}\left(-\dfrac{2l_j f''_{\mathrm{M}}(m_j^*)}{f'_{\mathrm{M}}(m_j^*)^3} + \dfrac{2l_j^2 m_j^* f''_{\mathrm{M}}(m_j^*)}{f'_{\mathrm{M}}(m_j^*)^3} - \sum\limits_{k=\alpha}^{\infty}\dfrac{l_k^2}{f'_{\mathrm{M}}(m_k^*)^2}\right) & i = j \\ \dfrac{\delta}{T^*}\dfrac{2l_i^2 m_j^* f''_{\mathrm{M}}(m_i^*)}{f'_{\mathrm{M}}(m_i^*)^3} & i \neq j. \end{cases} \tag{SI.90}$$

At any equilibrium of the form in Eq. (SI.36), Eqs. (SI.1) and (SI.36) imply that there is some constant $B_{\mathrm{M}}^* > 0$ independent of age j such that

$$\frac{l_j}{m_j^* f'(m_j^*)^2} = B_{\mathrm{M}}^*, \quad j = \alpha, \alpha+1\ldots \tag{SI.91}$$

Using this and the fact that $R^* = \sum_{k=\alpha}^{\infty} l_k m_k^* = 1$, we can rewrite Eq. (SI.90) as

$$\left.\frac{\partial F_j}{\partial m_i}\right|_{(m_n)=(m_n^*)} = \begin{cases} 1 + \dfrac{\delta}{T^*}\left(-\dfrac{2B_{\mathrm{M}}^* m_j^* f''_{\mathrm{M}}(m_j^*)}{f'_{\mathrm{M}}(m_j^*)} + \dfrac{2l_j B_{\mathrm{M}}^*(m_j^*)^2 f''_{\mathrm{M}}(m_j^*)}{f'_{\mathrm{M}}(m_j^*)} - B_{\mathrm{M}}^*\right) & i = j \\ \dfrac{\delta}{T^*}\dfrac{2l_i B_{\mathrm{M}}^* m_i^* m_j^* f''_{\mathrm{M}}(m_i^*)}{f'_{\mathrm{M}}(m_i^*)} & i \neq j. \end{cases} \tag{SI.92}$$

By weak selection ($0 < \delta \ll 1$) and the fact that $f_{\mathrm{M}}(m)$ solves Eq. (SI.87), this derivative is always positive. Define

$$\Delta^* m_j(t) = m_j(t) - m_j^*, \qquad j = \alpha, \alpha+1, \dots \tag{SI.93}$$

which gives the difference at t between m_j, $j = \alpha, \alpha+1, \dots$ and its equilibrium value. Let $(\Delta^* m_n(t)) = \Delta^* m_\alpha(t), \Delta^* m_{\alpha+1}(t), \dots$ be the corresponding sequence. Using these quantities and the map in Eq. (SI.89),

$$m_j^* + \Delta^* m_j(t+1) = F_j((m_n^*) + (\Delta^* m_n(t))), \qquad j = \alpha, \alpha+1, \dots \tag{SI.94}$$

Suppose $(m_n(t))$ is not at equilibrium but it is found in a sufficiently small neighbourhood of (m_n^*) so that we can linearly approximate the right hand side of Eq. (SI.94) as follows

$$\begin{aligned} F_j((m_n^*) + (\Delta^* m_n(t))) &\approx F_j((m_n^*)) + \sum_{i=\alpha}^{\infty} \Delta^* m_j(t) \left.\frac{\partial F_j}{\partial m_i}\right|_{(m_n)=(m_n^*)} \\ &= m_j^* + \sum_{i=\alpha}^{\infty} \Delta^* m_j(t) \left.\frac{\partial F_j}{\partial m_i}\right|_{(m_n)=(m_n^*)}, \quad j = \alpha, \alpha+1, \dots, \end{aligned} \tag{SI.95}$$

Substituting this last line into the right hand side of Eq. (SI.94) and simplifying we get

$$\Delta^* m_j(t+1) \approx \sum_{i=\alpha}^{\infty} \Delta^* m_j(t) \left.\frac{\partial F_j}{\partial m_i}\right|_{(m_n)=(m_n^*)}, \quad j = \alpha, \alpha+1, \dots, \tag{SI.96}$$

which gives, up to a linear approximation, the dynamics of the difference between $(m_n(t))$ and its equilibrium value around a small neighbourhood of this. Let

$$0 < \epsilon = \sup_n (|\Delta^* m_n(t)|), \tag{SI.97}$$

where we have the sequence $(|\Delta^* m_n(t)|) = |\Delta^* m_\alpha(t)|, |\Delta^* m_{\alpha+1}(t)|, \dots$. Upon taking absolute values of both sides of Eq. (SI.96), using Eq. (SI.97), the triangle inequality and recalling that the derivative in Eq. (SI.92) is always positive, we have

$$|\Delta^* m_j(t+1)| \le \epsilon \sum_{i=\alpha}^{\infty} \left.\frac{\partial F_j}{\partial m_i}\right|_{(m_n)=(m_n^*)}, \quad j = \alpha, \alpha+1, \dots \tag{SI.98}$$

Look at the series on the right hand side of Eq. (SI.98) and only consider solutions of the differential equation (SI.87). Using the fact that $R^* = 1$, we can see that

$$\begin{aligned} \sum_{i=\alpha}^{\infty} \left.\frac{\partial F_j}{\partial m_i}\right|_{(m_n)=(m_n^*)} &= 1 + \frac{\delta}{T^*} \left(-2c m_i^* B_{\mathrm{M}}^* + 2c m_i^* B_{\mathrm{M}}^* \sum_{k=\alpha}^{\infty} l_k m_k^* - B_{\mathrm{M}}^* \right) \\ &= 1 - \frac{\delta B_{\mathrm{M}}^*}{T^*}. \end{aligned} \tag{SI.99}$$

Let

$$0 < r = 1 - \frac{\delta B_{\mathrm{M}}^*}{T^*} < 1, \tag{SI.100}$$

where the inequalities derive from the assumption of weak selection ($0 < \delta \ll 1$). Rewriting Eq. (SI.98),

$$|\Delta^* m_j(t+1)| \le \epsilon r, \quad j = \alpha, \alpha+1, \dots \tag{SI.101}$$

which implies that

$$\sup_n (|\Delta^* m_n(t+1)|) \le \epsilon r \tag{SI.102}$$

Then, iterating the reasoning above for the next time step

$$|\Delta^* m_j(t+2)| \approx \left| \sum_{i=\alpha}^{\infty} \Delta^* m_j(t+1) \frac{\partial F_j}{\partial m_i}\bigg|_{(m_n)=(m_n^*)} \right| \leq \epsilon r \sum_{i=\alpha}^{\infty} \left| \frac{\partial F_j}{\partial m_i}\bigg|_{(m_n)=(m_n^*)} \right| = \epsilon r^2, \quad j = \alpha, \alpha+1, \dots \tag{SI.103}$$

By induction,

$$\sup_n(|\Delta^* m_n(t+k)|) \leq \epsilon r^k. \tag{SI.104}$$

Since

$$\lim_{k\to\infty} \sum_{i=\alpha}^{\infty} \frac{1}{2^i} \frac{\epsilon r^k}{1+\epsilon r^k} = \lim_{k\to\infty} \frac{2^{1-\alpha}\epsilon r^k}{1+\epsilon r^k} = 0, \tag{SI.105}$$

using Eq. (SI.7), we can see from Eqs. (SI.93) and (SI.104) that

$$\lim_{t\to\infty} d_\alpha((m_n(t)), (m_n^*)) = 0. \tag{SI.106}$$

Therefore, when $(m_n(t))$ is within a sufficiently small neighbourhood of (m_n^*), the distance between $(m_n(t))$ and the equilibrium eventually goes to zero under the dynamics in Eq. (SI.89). An equilibrium (m_n^*) of the form in Eq. (SI.36), if it exists for a given solution of Eq. (SI.87), is linearly stable.

2.5.7 Instability of negative ageing in fecundity

Let $f_\mathrm{M} \in \mathscr{F}_-^m$. If an equilibrium (m_n^*) of the form in Eq. (SI.36) exists, there is negative ageing in fecundity. Using the same approach as in Section 2.5.5, the special case $f_\mathrm{M}(m) = m^q$ with $0 < q < 1/2$ leads to $\Delta V^m(t) > 0$ in Eq. (SI.85). Therefore, if there were an equilibrium (m_n^*) when $f_\mathrm{M}(m) = m^q$ with $0 < q < 1/2$, this would be unstable.

But negative ageing can be shown unstable in general when $f_\mathrm{M} \in \mathscr{F}_-^m$. Using Eq. (SI.27) and with a first order Taylor expansion around $\delta = 0$, it is possible to get the following dynamical system

$$\frac{m_j(t+1)}{m_\alpha(t+1)} \approx \frac{m_j(t)}{m_\alpha(t)} + \delta \frac{l_\alpha}{T(t) m_\alpha(t) f'_\mathrm{M}(m_j(t))^2} \left[\frac{l_j}{l_\alpha} - \frac{m_j(t) f'_\mathrm{M}(m_j(t))^2}{m_\alpha(t) f'_\mathrm{M}(m_\alpha(t))^2} \right], \quad j = \alpha+1, \alpha+2, \dots \tag{SI.107}$$

Using Eq. (SI.107), we can study the dynamics of the sequence $m_{\alpha+1}, m_{\alpha+2}, \dots$ relative to the dynamics of m_α. Imagine an equilibrium (m_n^*) exists for the given $f_\mathrm{M} \in \mathscr{F}_-^m$. Consider the dynamics of the system in Eq. (SI.107) in the subset of the space that is obtained by setting fecundity at age α equal to its equilibrium value at all time, so that $m_\alpha(t) = m_\alpha^*$ for all t, i.e. no dynamics of m_α. Let $(m_n)_{\alpha+1}$ be the subsequence $m_{\alpha+1}, m_{\alpha+2}, \dots$. Setting $m_\alpha(t) = m_\alpha^*$ into Eq. (SI.107) and multiplying through by m_α^*, we obtain

$$m_j(t+1) = m_j(t) + \delta \underbrace{\frac{l_\alpha}{m_\alpha^* f'_\mathrm{M}(m_\alpha^*)^2 T(t) f'_\mathrm{M}(m_j(t))^2}}_{:=c^m_{j,\alpha}(t)} \left[\underbrace{\frac{m_\alpha^* f'_\mathrm{M}(m_\alpha^*)^2 l_j}{l_\alpha}}_{:=w_j} - m_j(t) f'_\mathrm{M}(m_j(t))^2 \right], \quad j = \alpha+1, \alpha+2, \dots, \tag{SI.108}$$

which gives the dynamics of $(m_n)_{\alpha+1}$ when m_α is held constant at equilibrium value and where $c^m_{j,\alpha}(t) > 0$, $j = \alpha+1, \alpha+2, \dots$, independently of t and w_j, $j = \alpha+1, \alpha+2, \dots$, is a positive constant. By Eq. (SI.42), there exists a function $I_m(z)$ that is the inverse function of $mf'(m)^2$ and both $I_m(z)$ and $mf'(m)^2$ invert order on $(0, \infty)$. The quantity in brackets in Eq. (SI.108) shows that at equilibrium,

$$m_j^* = I_m(w_j), \quad j = \alpha+1, \alpha+2, \dots \tag{SI.109}$$

Form the sequence $(I_m(w_n))_{\alpha+1} = I_m(w_{\alpha+1}), I_m(w_{\alpha+2}), \dots$. Suppose that $(m_n)_{\alpha+1}$ is not at equilibrium at t. Using Eq. (SI.7), its distance from the equilibrium is

$$d_{\alpha+1}((m_n(t))_{\alpha+1}, (I_m(w_n))_{\alpha+1}) = \sum_{j=\alpha+1}^{\infty} \frac{1}{2^j} \frac{|m_j(t) - I_m(w_j)|}{1 + |m_j(t) - I_m(w_j)|} > 0. \tag{SI.110}$$

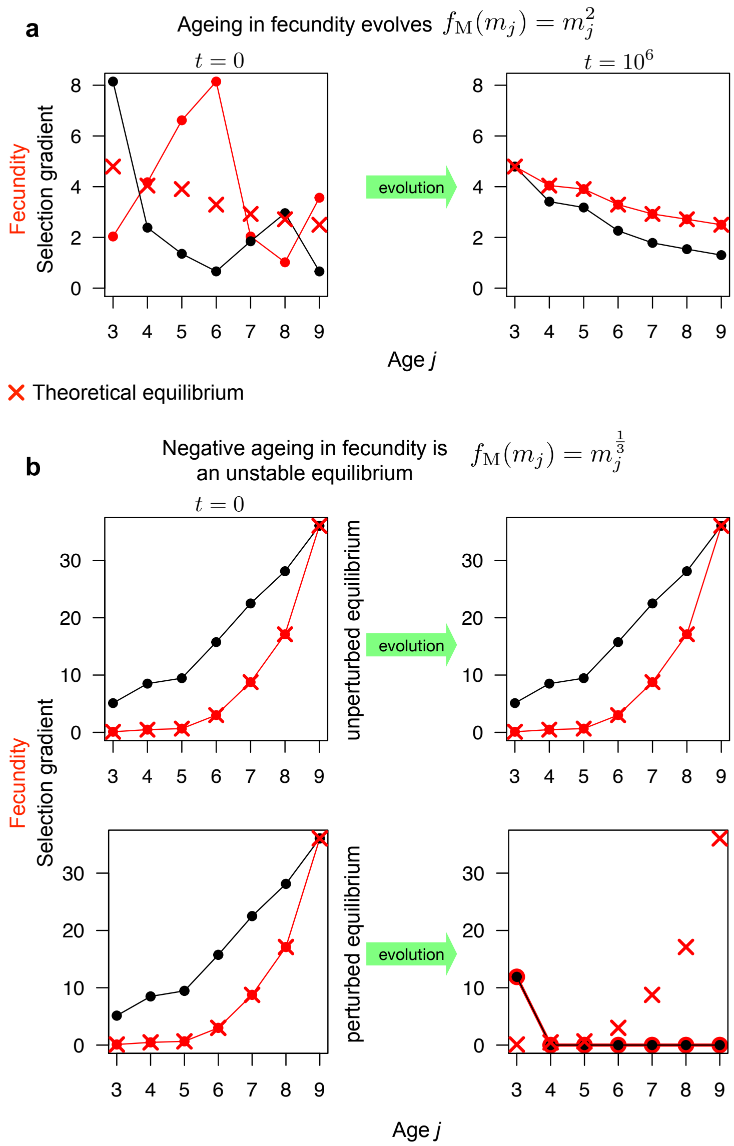

Figure SI.2: **Evolution of fecundity.** The evolution of fecundity under the dynamics in Eq. (SI.27) is explored for two different functions f_M, see Section 2.3, while imposing a maximum age ω. Survival is constant with p_0=0.7, p_1=0.3, p_2=0.4, p_3=0.6, p_4=0.9, p_5=0.6, p_6=0.7, p_7=0.8 and p_8=0.78 with ω=9. Initial and final fecundity levels as well as selection gradients are reported. For visualisation, selection gradients are scaled so that their maximum value coincides with the maximum fecundity. **a** Ageing in fecundity evolves. Stability of ageing in fecundity is shown for a function in $\mathscr{F}_{+}^{m}$, which implies an equilibrium life history with ageing in fecundity (Section 2.3). Starting from far away, the life history eventually gets to the equilibrium predicted by Eq. (SI.47). The parameter δ (additive genetic variance) in Eq. (SI.27) is set equal to 0.1. Initial fecundities are chosen so that, at demographic stability, the life history generates a demographically stationary population. Equilibrium values are found via Eq. SI.47 by imposing the maximum age $\omega = 9$. **b** Negative ageing in fecundity is an unstable equilibrium. Instability of negative ageing in fecundity is shown for a function in $\mathscr{F}_{-}^{m}$, which implies an equilibrium life history with negative ageing in fecundity (Section 2.3). The initial fecundity schedule is set at the equilibrium predicted by Eq. (SI.51). If unperturbed, this equilibrium is maintained. If perturbed, the life history goes away from it and asymptotically tends to a form of semelparity (i.e. single reproductive event). The one-time perturbation consists in adding 0.001 and -0.001 to fecundity at odd and even ages, respectively, to equilibrium values and then scaling fecundity to ensure demographic stationarity. The parameter δ (additive genetic variance) in Eq. (SI.27) is set equal to 0.001. Equilibrium values are found via Eq. (SI.51) by imposing maximum age ω=9.

Let $K \subset \{\alpha+1, \alpha+2, \dots\}$ be the index set such that $j \in K$ if and only if the term with subscript j in the series given by $d_{\alpha+1}((m_n(t))_{\alpha+1}, (I_m(w_n))_{\alpha+1})$ is positive. The set K is not empty, as $(m_n)_{\alpha+1}$ is not at equilibrium at t.

If $m_j(t) > I_m(w_j)$ for some $j = K$, then $m_j(t)f'(m_j(t))^2 < w_j, \quad j = K$. By Eq. (SI.108), this in turn implies that $m_j(t+1) > m_j(t) > I_m(w_j)$. Hence,

$$\text{If } m_j(t) > I_m(w_j), \text{ then } |m_j(t+1) - I_m(w_j)| > |m_j(t) - I_m(w_j)|, \quad j = K \tag{SI.111}$$

If $m_j(t) < I_m(w_j)$ for some $j = K$, then $m_j(t)f'(m_j(t))^2 > w_j, \quad j = K$. By Eq. (SI.108), this in turn implies that $m_j(t+1) < m_j(t) < I_m(w_j)$. Hence,

$$\text{If } m_j(t) < I_m(w_j), \text{ then } |m_j(t+1) - I_m(w_j)| > |m_j(t) - I_m(w_j)|, \quad j = K \tag{SI.112}$$

Therefore,

$$\text{If } m_j(t) \neq I_m(w_j), \text{ then } |m_j(t+1) - I_m(w_j)| > |m_j(t) - I_m(w_j)|, \quad j = K \tag{SI.113}$$

Moreover, the dynamics in Eq. (SI.108) also show that, when $K \subset \{\alpha+1, \alpha+2, \dots\}$ but $K \neq \{\alpha+1, \alpha+2, \dots\}$,

$$\text{If } m_j(t) = I_m(w_j), \text{ then } |m_j(t+1) - I_m(w_j)| = |m_j(t) - I_m(w_j)| = 0, \quad j \in \{\alpha+1, \alpha+2, \dots\} \setminus K \tag{SI.114}$$

From Eqs. (SI.113)-(SI.114), for any term equal to zero in the series given by $d_{\alpha+1}((m_n(t))_{\alpha+1}, (I_m(w_n))_{\alpha+1})$, the corresponding term in the series given by $d_{\alpha+1}((m_n(t+1))_{\alpha+1}, (I_m(w_n))_{\alpha+1})$ is still equal to zero, while all positive terms in the series given by $d_{\alpha+1}((m_n(t))_{\alpha+1}, (I_m(w_n))_{\alpha+1})$ are smaller than the corresponding terms in the series given by $d_{\alpha+1}((m_n(t+1))_{\alpha+1}, (I_m(w_n))_{\alpha+1})$. Thus, using Eq. (SI.109),

$$d_{\alpha+1}((m_n(t+1))_{\alpha+1}, (m_n^*)_{\alpha+1}) > d_{\alpha+1}((m_n(t))_{\alpha+1}, (m_n^*)_{\alpha+1}) > 0, \tag{SI.115}$$

where $(m_n^*)_{\alpha+1} = m_{\alpha+1}^*, m_{\alpha+2}^*, \dots$ The dynamics of $(m_n(t))_{\alpha+1}$ always repel equilibrium, because when $(m_n)_{\alpha+1}$ is at any positive distance from it, this distance will keep increasing with time. Any equilibrium $(m_n^*)_{\alpha+1}$ and, consequently, any equilibrium (m_n^*) is unstable when $f_{\mathrm{M}} \in \mathscr{F}_-^m$. Note that the choice of keeping m_α fixed at its equilibrium value is arbitrary. We could have chosen any other fecundity to be kept at equilibrium value and, with the due modifications, the same argument about the repellence of the equilibrium with negative ageing would hold.

2.6 Numerical solutions

The dynamics of the original system in Eq. (SI.27) were simplified via the introduction of a maximum age ω, i.e. $p_\omega = 0$. These simplified dynamics were numerically explored to support analytic results (Fig. SI.2). Stability of the equilibrium life history with ageing in fecundity predicted from Eq. (SI.47) is supported for a choice of $f_{\mathrm{M}} \in \mathscr{F}_+^m$. For a choice of $f_{\mathrm{M}} \in \mathscr{F}_-^m$, the equilibrium with negative ageing derived from Eq. (SI.51) is shown unstable. Slightly perturbing this equilibrium, the life history goes away from it.

3 Analysis of survival evolution

The analysis of evolutionary dynamics of fecundity has some analogies with that of fecundity. However, all arguments are here given in full for ease of reading. As in Section 2, asterisks denote equilibrium quantities.

3.1 Equilibria in survival evolution

Here some general properties of equilibria in survival evolution are derived. To this aim, let us linearly approximate the quantity $\Delta_s\lambda$ in Eq. (SI.33) with the total differential $\mathrm{d}\lambda$. Define $\Delta_s f_P(p_j) = \frac{\delta}{T}\frac{\partial v_0}{\partial f_P(p_i)}$ as the change after selection, but before rescaling, see Eq. (SI.30). With the assumption of demographic stationarity, $\lambda = 1$, the discussion in Section 1.4 and using Eq. (SI.25), one has that

$$\mathrm{d}\lambda = \sum_{i=0}^{\infty} \frac{\partial\lambda}{\partial f_P(p_i)}\Delta_s f_P(p_i) = \sum_{i=0}^{\infty} \frac{\partial\lambda}{\partial f_P(p_i)}\left(\frac{\delta}{T}\frac{\partial v_0}{\partial f_P(p_i)}\right) = \delta \sum_{i=0}^{\infty}\left(\frac{H_i}{p_i f'_P(p_i)}\right)^2, \qquad \text{(SI.116)}$$

where the series is assumed to converge because, in virtue of weak selection ($0 < \delta \ll 1$), the quantity $\Delta_s\lambda \approx \mathrm{d}\lambda$ is small. Using $\mathrm{d}\lambda$ instead of $\Delta_s\lambda$ in Eq. (SI.33), a first-order Taylor expansion around $\delta = 0$ in Eq. (SI.33) yields

$$p_j(t+1) \approx p_j(t)\left[1 + \delta\left(\frac{H_j(t)}{p_j^2(t) f'_P(p_j(t))^2} - \sum_{i=0}^{\infty}\frac{H_i^2(t)}{p_i^2(t) f'_P(p_i(t))^2}\right)\right], \qquad j = 0, 1, \ldots, \qquad \text{(SI.117)}$$

where the general relationship $(f^{-1}(z))' = 1/f'(f^{-1}(z))$ was used. From Eq. (SI.22) and (SI.117), equilibrium survival, i.e. $p_j(t+1) = p_j(t)$ for all j, implies

$$\frac{H_j^*}{(p_j^*)^2 f'_P(p_j^*)^2} = \frac{H_k^*}{(p_k^*)^2 f'_P(p_k^*)^2} \quad \text{for} \quad j, k = 0, 1, \ldots, \qquad \text{(SI.118)}$$

where * denotes quantities in equilibrium. For our purposes, it is more convenient to write Eq. (SI.118) as a pairwise comparison at two successive ages,

$$\frac{\sqrt{H_{j+1}^*}}{p_{j+1}^* f'_P(p_{j+1}^*)} = \frac{\sqrt{H_j^*}}{p_j^* f'_P(p_j^*)}, \qquad j = 0, 1, \ldots \qquad \text{(SI.119)}$$

From these equilibrium equations and Eq. (SI.21), one obtains

$$p_{j+1}^* f'_P(p_{j+1}^*) = p_j^* f'_P(p_j^*), \qquad j = 0, \ldots, \alpha - 2 \qquad \text{(SI.120a)}$$

$$p_{j+1}^* f'_P(p_{j+1}^*) < p_j^* f'_P(p_j^*), \qquad j = \alpha - 1, \alpha, \ldots \qquad \text{(SI.120b)}$$

Constant survival at pre-reproductive ages is always an equilibrium, while a flat trajectory of survival over reproductive ages cannot be an equilibrium, as $p_{j+1}^* = p_j^*$, $j = \alpha - 1, \alpha, \ldots$, implies a contradiction to Eq. (SI.120b).

3.2 Equilibrium selection gradient on survival

Multiplying Eq. (SI.119) by $\sqrt{H_{j+1}^*}$, one obtains

$$\frac{H_{j+1}^*}{p_{j+1}^* f'_P(p_{j+1}^*)} = \frac{\sqrt{H_j^*}\sqrt{H_{j+1}^*}}{p_j^* f'_P(p_j^*)} \leq \frac{\sqrt{H_j^*}\sqrt{H_j^*}}{p_j^* f'_P(p_j^*)} = \frac{H_j^*}{p_j^* f'_P(p_j^*)}, \quad j = 0, 1, \ldots, \qquad \text{(SI.121)}$$

where the inequality arises from Eq. (SI.21). More explicitly, we have

$$\frac{H_{j+1}^*}{p_{j+1}^* f'_P(p_{j+1}^*)} = \frac{H_j^*}{p_j^* f'_P(p_j^*)}, \qquad j = 0, \ldots, \alpha - 2, \qquad \text{(SI.122a)}$$

$$\frac{H_{j+1}^*}{p_{j+1}^* f'(p_{j+1}^*)} < \frac{H_j^*}{p_j^* f'_P(p_j^*)}, \qquad j = \alpha - 1, \alpha, \ldots \qquad \text{(SI.122b)}$$

A look at Eq. (SI.25a) reveals that Eqs. (SI.122) describes the equilibrium selection gradient on age-specific survival, which is flat at pre-reproductive ages, while it decreases in magnitude with increasing reproductive age. This equilibrium result is independent of the choice of f_P.

3.3 Sets of functions for survival evolution

Aside from the restriction of having a positive first derivative, the function f_{P} requires further specification. Here two sets of such functions are defined. Each set contains functions that will be shown to lead to qualitatively similar dynamical behaviour. Consider the differential equation

$$(pf'(p))' = f'(p) + pf''(p) = 0, \quad p \in (0,1). \tag{SI.123}$$

The general solution of this differential equation is $f(p) = a_1 \ln p + a_2$, where a_1 and a_2 are constants. To ensure that the first derivative on $(0,1)$ is positive, we need $a_1 > 0$. The parametrization of the selection gradient in the classic theory (i.e. $f_{\mathrm{P}}(p_j) = \ln p_j$) [11] is a particular solution to Eq. (SI.123).

But if f_{P} fulfills Eq. (SI.123), then $pf'_{\mathrm{P}}(p)$ takes a constant value for any $p \in (0,1)$. Therefore, solutions of Eq. (SI.123) lead to a contradiction with the equilibrium in Eq. (SI.120b) for reproductive ages. This result naturally leads to consider two disjoint sets of functions to parametrize the selection gradient on survival. These sets are obtained by looking at the differential inequalities corresponding to the differential equation in Eq. (SI.123).

3.3.1 Definition of $\mathscr{F}^p_+$

The first set is

$$\mathscr{F}^p_+ := \{f_{\mathrm{P}} : (pf'_{\mathrm{P}}(p))' > 0 \text{ for } p \in (0,1)\}. \tag{SI.124}$$

From Eq. (SI.123), linear or convex (i.e. $f''_{\mathrm{P}}(p) \geq 0$) functions on $(0,1)$ with positive first derivative form a subset of $\mathscr{F}^p_+$. But also some concave functions belong to $\mathscr{F}^p_+$. All power functions $a_1 y^q + a_2$ with $a_1 >$ and $q > 0$ are in $\mathscr{F}^p_+$, although these functions are concave for $0 < q < 1$. Examples of other elementary functions in $\mathscr{F}^p_+$ are $\exp(p)$, $\tan(p)$, $-\ln(1-p)$ and $-\ln(-\ln p)$. The fact that the last two functions are in $\mathscr{F}^p_+$ is relevant because when p is age-specific survival, they indicate that survival evolution proceeds by multiplicative decrements in the age-specific probability of death and in the age-specific average mortality, respectively [13]. It is plausible that, in some cases, mutations have additive effects on this transformation of age-specific survival [13, 15]. By strict monotonicity

$$\text{if } f_{\mathrm{P}} \in \mathscr{F}^p_+, \text{ then } pf'_{\mathrm{P}}(p) \text{ preserves order on } (0,1) \tag{SI.125}$$

and functions $pf'_{\mathrm{P}}(p)$ are invertible on $(0,1)$.

3.3.2 Definition of $\mathscr{F}^p_-$

The second set of functions is

$$\mathscr{F}^p_- := \{f_{\mathrm{P}} : (pf'_{\mathrm{P}}(p))' > 0 \text{ for } p \in (0,b) \text{ and } (pf'_{\mathrm{P}}(p))' < 0 \text{ for } p \in (b,1)\} \tag{SI.126}$$

for some $0 < b < 1$, which then is the only root of $f'_{\mathrm{P}}(p) + pf''_{\mathrm{P}}(p)$ on $(0,1)$. Let $f_{\mathrm{P}} \in \mathscr{F}^p_-$. Then, as $f'_{\mathrm{P}}(p) > 0$, f_{P} must be somewhere concave, i.e. $f''_{\mathrm{P}}(p) < 0$ in $(b,1)$. Not all functions that are concave somewhere on $(0,1)$ are in $\mathscr{F}^p_-$. For example, p^q with $0 < q < 1$ is concave on $(0,1)$ yet it belongs to $\mathscr{F}^p_+$. Examples of elementary functions in $\mathscr{F}^p_-$ are $\sqrt{y}/(1+p)$ with $b \approx 0.1716$, $\arctan^{\frac{1}{\pi}}(p)$ with $b \approx 0.5122$ and $\sin(p)$ with $b \approx 0.8603$, where the b values are retrieved via the root finding function `optimize` in R (version 3.3.3). The splitting of the $(0,1)$ interval by b is introduced because, near zero, the sign of $f'_{\mathrm{P}}(p) + pf''_{\mathrm{P}}(p)$ is likely determined by the sign of the assumed positive $f'_{\mathrm{P}}(p)$ term, as in many relevant cases $pf''_{\mathrm{P}}(p)$ vanishes as p tends to 0. For analytical simplicity, only the cases in which b is either close to 1 or close to 0 are considered, so that only one of the inequalities in (SI.126) is truly relevant. By strict monotonicity

$$\text{if } f_{\mathrm{P}} \in \mathscr{F}^p_-, \text{ then } pf'_{\mathrm{P}}(p) \text{ preserves order on } (0,b) \text{ and inverts order on } (b,1) \tag{SI.127}$$

and functions $pf'_{\mathrm{P}}(p)$ are invertible in the relevant intervals.

3.4 Survival at equilibria

Equilibria of the form in Eq. (SI.119), if any exists for the given fecundities and the chosen f_{P}, obey a recursion. Note that equilibrium survival must satisfy both the equilibrium equations (SI.119) and Euler-Lotka equation (SI.2) at demographic stationarity.

Let us first focus on the equilibrium equations (SI.119), which can be expressed as

$$p_j^* f_{\mathrm{P}}'(p_j^*) = p_0^* f_{\mathrm{P}}'(p_0^*) \sqrt{\frac{H_j^*}{H_0^*}}, \quad j = 1, 2, \ldots \tag{SI.128}$$

An alternative way of expressing the ratio H_j^*/H_0^* using Eqs. (SI.2) and (SI.20) and the assumption of demographic stationarity ($\lambda = 1$) is

$$\frac{H_j^*}{H_0^*} = \sum_{i=j+1}^{\infty} l_i^* m_i = 1 - \sum_{i=1}^{j} l_i^* m_i, \quad j = 1, 2, \ldots \tag{SI.129}$$

where $l_i^* = p_0^* p_1^* \ldots p_{i-1}^*$. Let $p_0^* = u$ where u is in $(0,1)$. Let $P(z)$ be the inverse function of $z f_{\mathrm{P}}'(z)$. As equilibrium pre-reproductive survival is constant, $l_\alpha^* = p_0^* p_1^* ... p_{\alpha-1}^* = p_0^* p_0^* ... p_0^* = u^\alpha$. Using the function $P(z)$, the equilibrium equations in (SI.128) can be rewritten recursively in terms of u and the constants m_j, which we recall are $m_j = 0$ for $j = 1, 2, \ldots, \alpha - 1$,

$$\begin{aligned}
p_\alpha^*(u) &= P\left(p_0^* f_{\mathrm{P}}'(p_0^*) \sqrt{1 - \sum_{i=1}^{\alpha} l_i^*(u) m_i} \right) = P\left(u f_{\mathrm{P}}'(u) \sqrt{1 - u^\alpha m_\alpha} \right) \\
p_{\alpha+1}^*(u) &= P\left(p_0^* f_{\mathrm{P}}'(p_0^*) \sqrt{1 - \sum_{i=1}^{\alpha+1} l_i^*(u) m_i} \right) = P\left(u f_{\mathrm{P}}'(u) \sqrt{1 - u^\alpha m_\alpha - u^\alpha p_\alpha^*(u) m_{\alpha+1}} \right) \\
p_{\alpha+2}^*(u) &= P\left(u f_{\mathrm{P}}'(u) \sqrt{1 - u^\alpha m_\alpha - u^\alpha p_\alpha^*(u) m_{\alpha+1} - u^\alpha p_\alpha^*(u) p_{\alpha+1}^*(u) m_{\alpha+2}} \right) \\
&\vdots
\end{aligned} \tag{SI.130}$$

Let U be the set of values of u that satisfy all the equations (SI.130).

Next, let us write the Euler-Lotka equation (SI.2) at demographic stationarity ($\lambda = 1$) using equilibrium survival parametrized by u,

$$\sum_{i=1}^{\infty} l_i(u) m_i = 1. \tag{SI.131}$$

Suppose $u^* \in U$ solves this equation. Then u^* determines an equilibrium, as it solves both the Euler-Lotka equation and the equilibrium condition Eq. (SI.119).

Three things should be noted about the recursion above. First, U can be empty, e.g. some square root has no real solution for any $u \in (0,1)$. Second, it may be that no $u \in U$ is a solution of Eq. (SI.131). Therefore, the existence of an equilibrium for the given fecundities and f_{P} is not established. Third, the recursion is infinite, numerical in essence and it contains a function, $P(z)$, which may not have closed form. Hence, the recursion is of limited practical use. However, it still has theoretical value, as the next section shows.

3.4.1 Ageing in survival at equilibrium

Suppose that $f_{\mathrm{P}} \in \mathscr{F}_+^p$ and the system in Eq. (SI.117) has an equilibrium. By Eqs. (SI.120) and (SI.125),

$$p_{j+1}^* < p_j^*, \quad j = \alpha - 1, \alpha, \ldots, \qquad \text{with } f_{\mathrm{P}} \in \mathscr{F}_+^p. \tag{SI.132}$$

Therefore, any equilibrium life history shows ageing in survival.

A necessary condition for the existence of this equilibrium and for guaranteeing that equilibrium survival does not exceed 1 is that, if the power series

$$\sigma(u) = \sum_{i=1}^{\infty} u^i m_i. \tag{SI.133}$$

contains $(0,1)$ in its radius of convergence, then it takes value 1 for some value of u in $(0,1)$. If the power series $\sigma(u)$ converges on $(0,1)$, then it is differentiable on $(0,1)$, as any power series is differentiable within its radius of convergence. Suppose then that an equilibrium exists: there is some $u^* \in U$ that solves Eq. (SI.131). Since u^* equals age-specific survival at some pre-reproductive age, $u^* \in (0,1)$. As at this equilibrium there is ageing in survival, the inequality $l_i(u^*) \leq (u^*)^i$, $i = 0, 1, \ldots$, holds. Let then $\sigma_\triangle(u)$ be the series on the left hand side of Eq. (SI.131). Term-wise comparison shows that $\sigma(u^*) \geq \sigma_\triangle(u^*)$. As $\sigma'(u) > 0$ for $u > 0$ and $\sigma(0) = 0$, if the equation $\sigma(u) = 1$ has a solution for $u \in (0,1)$, then this solution is unique on this interval. Therefore, if $\sigma_\triangle(u) = 1$ has a solution in the interval $(0,1)$, then the unique root of $\sigma(u) = 1$ is also in the interval $(0,1)$. By contraposition, if $\sigma(u) = 1$ has no root in $(0,1)$, then $\sigma_\triangle(u) = 1$ also fails to have a root in $(0,1)$.

A sufficient condition to ensure an equilibrium with ageing in survival is given in Section 3.7.

3.4.2 Negative ageing in survival at equilibrium

Suppose that $f_\mathrm{P} \in \mathscr{F}^p_-$ and the system in Eq. (SI.117) has an equilibrium. If $p^*_j > b$ for $j = \alpha - 1, \alpha, \ldots$ (where b is as defined in Section 3.3.2), then, by Eqs. (SI.120) and (SI.127), $p^*_{j+1} > p^*_j$ for $j = \alpha - 1, \alpha, \ldots$. Therefore, an equilibrium life history might show negative ageing in survival. If $p^*_j < b$ for $j = \alpha - 1, \alpha, \ldots$, then $p^*_{j+1} < p^*_j$ for $j = \alpha - 1, \alpha, \ldots$ and ageing in survival is again an equilibrium. For analytic simplicity, the case in which some p^*_j are greater than b and some other p^*_j are smaller is not considered.

3.5 Dynamical properties of survival evolution

3.5.1 Coordinate transformation

Some key dynamical properties of survival evolution are better understood with a coordinate transformation. The original system in Eq. (SI.33) has variables $p_0, p_1, \ldots$, i.e. a sequence (p_n), which must satisfy the Euler-Lotka equation (SI.2) at all times $t = 0, 1, \ldots$ for $\lambda = 1$ and the given fecundities. Consider then the transformed system in the variables

$$x_j := \frac{p_{j+1}}{p_j}, \quad j = 0, 1, \ldots \tag{SI.134}$$

which form another sequence $(x_n) = x_0, x_1, \ldots$ that is defined as long as $p_j > 0$ for $j = 0, 1, \ldots$. In this regard, recall that the positivity of survival is guaranteed by the dynamics in Eq. (SI.33) as long as survival in the system evolution does not exceed 1, as assumed. The transformation in Eq. (SI.134) is invertible. Let $p_0 = u$, where u is an unknown real number in $(0,1)$. Note that

$$p_k = u \prod_{i=0}^{k-1} x_i, \quad k = 0, 1, \ldots \tag{SI.135}$$

Hence, each single value of u uniquely determines the sequence (p_n). From Eq. (SI.1),

$$\begin{aligned} l_j = \prod_{k=0}^{j-1} p_k &= u \left(u \prod_{i=0}^{0} x_i \right) \left(u \prod_{i=0}^{1} x_i \right) \cdots \left(u \prod_{i=0}^{j-2} x_i \right) \\ &= u^j \prod_{k=0}^{j-2} \prod_{i=0}^{k} x_i, \quad j = 0, 1, \ldots \end{aligned} \tag{SI.136}$$

One can use Eq. (SI.136) and the given fecundities to write the left hand side of Euler-Lotka equation (SI.2) with $\lambda = 1$ as a function β of u given by a power series,

$$\beta(u) = \sum_{j=1}^{\infty} u^j \left(\prod_{k=0}^{j-2} \prod_{i=0}^{k} x_i \right) m_j. \tag{SI.137}$$

which, being the left hand side of Euler-Lotka equation, must converge to 1 for some value of u in $(0,1)$. Suppose $\beta(u)$ converges to 1 for two distinct values $\hat{u}_1$ and $\hat{u}_2$ of u in $(0,1)$. Then, the interval $(\hat{u}_1, \hat{u}_2)$ is both within $(0,1)$ and within the radius of convergence of $\beta(u)$. Any power series is differentiable within the radius of convergence. Since $\beta'(u) > 0$ in $(\hat{u}_1, \hat{u}_2)$, we have that $\hat{u}_1$ and $\hat{u}_2$ cannot both be roots of $\beta(u) = 1$. Therefore, there is a unique value $\hat{u}$ of u in $(0,1)$ such that $\beta(\hat{u}) = 1$. Hence, to the sequence $x_0, x_1, \dots$ there corresponds corresponds a unique value $p_0 = \hat{u}$ for the first term of the original sequence (p_n). Via Eq. (SI.135), the whole original sequence (p_n) of survival probabilities is uniquely determined and, therefore, the transformation is invertible. In the following analysis of the transformed system, the quantities p_j, $j = 0, 1, \dots$, should be seen as functions of the sequence (x_n), as seen above.

3.5.2 Dynamics of the transformed system

Taking ratios of the equations of the system in Eq. (SI.33) and with a first order Taylor expansion around $\delta = 0$, the dynamics of the transformed system are given by equations of the form

$$\begin{aligned} x_j(t+1) &= \frac{f_{\mathrm{P}}^{-1}\left(f_{\mathrm{P}}(p_{j+1}(t)) + \delta \frac{1}{f'_{\mathrm{P}}(p_{j+1}(t))} \frac{H_{j+1}(t)}{p_{j+1}(t)}\right)}{f_{\mathrm{P}}^{-1}\left(f_{\mathrm{P}}(p_j(t)) + \delta \frac{1}{f'_{\mathrm{P}}(p_j(t))} \frac{H_j(t)}{p_j(t)}\right)} \\ &\approx x_j(t) + \delta \frac{1}{p_j(t)^2} \left[\frac{p_j(t)}{p_{j+1}(t)} \frac{H_{j+1}(t)}{f'(p_{j+1}(t))^2} - \frac{p_{j+1}(t)}{p_j(t)} \frac{H_j(t)}{f'(p_j(t))^2} \right] \\ &= x_j(t) + \delta c_j^p(t) \left[\frac{H_{j+1}(t)}{H_j(t)} - \psi_j(t) \right], \quad j = 0, 1 \dots \end{aligned} \tag{SI.138}$$

where

$$c_j^p(t) = \frac{H_j(t)}{p_j(t) p_{j+1}(t) f'_{\mathrm{P}}(p_{j+1}(t))^2} > 0 \tag{SI.139}$$

and

$$\psi_j(t) = \left(\frac{p_{j+1}(t) f'_{\mathrm{P}}(p_{j+1}(t))}{p_j(t) f'_{\mathrm{P}}(p_j(t))} \right)^2. \tag{SI.140}$$

Here, $c_j^p(t)$ always is greater than zero because it is a product of positive quantities. Supposing that $x_j(t) > 0$, $j = 0, 1, \dots$, for all t, the system in Eq. (SI.138) can always be made correspond to the original system in Eq. (SI.33), as shown in the previous section. Working with Eq. (SI.138) seems complicated because of the laborious evaluation of $p_0, p_1, \dots$, which are here each a function of the sequence (x_n). However, the transformed system can still help us to gain theoretical understanding of the original system as the next sections will show.

3.5.3 Basic properties of the transformed system

Here are some basic properties connecting x_j and ψ_j, $j = 0, 1, \dots$. From Eqs. (SI.125) and (SI.127), one has that

$$x_j > 1 \quad \text{if and only if} \quad \psi_j > 1, \qquad f_{\mathrm{P}} \in \mathscr{F}_+^p \text{ or } f_{\mathrm{P}} \in \mathscr{F}_-^p \text{ and } p_j, p_{j+1} < b \tag{SI.141a}$$

$$x_j < 1 \quad \text{if and only if} \quad \psi_j < 1, \qquad f_{\mathrm{P}} \in \mathscr{F}_+^p \text{ or } f_{\mathrm{P}} \in \mathscr{F}_-^p \text{ and } p_j, p_{j+1} < b. \tag{SI.141b}$$

From Eq. (SI.127), we find

$$x_j > 1 \quad \text{if and only if} \quad \psi_j < 1, \qquad f_{\mathrm{P}} \in \mathscr{F}_-^p \text{ and } p_j, p_{j+1} > b \tag{SI.142a}$$

$$x_j < 1 \quad \text{if and only if} \quad \psi_j > 1, \qquad f_{\mathrm{P}} \in \mathscr{F}_-^p \text{ and } p_j, p_{j+1} > b. \tag{SI.142b}$$

From Eqs. (SI.125) and (SI.127), we obtain

$$x_j = 1 \quad \text{if and only if} \quad \psi_j = 1, \qquad f_{\mathrm{P}} \in \mathscr{F}_+^p \text{ or } f_{\mathrm{P}} \in \mathscr{F}_-^p \text{ and either } p_j, p_{j+1} > b \text{ or } p_j, p_{j+1} < b. \tag{SI.143}$$

Further properties connecting x_j and ψ_j are derived by noting that any given positive value of x_j corresponds to the slope of a line through the origin in the p_j-p_{j+1} plane. Let θ_j be the angle between the (horizonal) p_j-axis and the line from the origin that goes through the point (p_j, p_{j+1}), which lies in a circle of radius ϱ_j.

Let us then switch to polar coordinates, $\psi_j(p_j, p_{j+1}) = \psi_j(\theta_j, \varrho_j)$, where $\varrho_j = \sqrt{p_j^2 + p_{j+1}^2}$ and $\theta_j = \arctan\left(\frac{p_{j+1}}{p_j}\right) = \arctan x_j$, such that $p_{j+1} = \varrho_j \sin(\theta_j)$ and $p_j = \varrho_j \cos(\theta_j)$. Differentiating with respect to the angle, one has that

$$\begin{aligned}
\frac{\partial \psi_j}{\partial \theta_j} &= \frac{\partial p_{j+1}}{\partial \theta_j}\frac{\partial \psi_j}{\partial p_{j+1}} + \frac{\partial p_j}{\partial \theta_j}\frac{\partial \psi_j}{\partial p_j} \\
&= p_j \frac{\partial \psi_j}{\partial p_{j+1}} - p_{j+1}\frac{\partial \psi_j}{\partial p_j} \\
&= 2\sqrt{\psi_j}\left[\frac{1}{f'_{\mathrm{P}}(p_j)}\frac{\partial}{\partial p_{j+1}}(p_{j+1} f'_{\mathrm{P}}(p_{j+1})) - p_{j+1}^2 f'_{\mathrm{P}}(p_{j+1})\frac{\partial}{\partial p_j}\frac{1}{(p_j f'_{\mathrm{P}}(p_j))}\right] \\
&= 2\sqrt{\psi_j}\left[\frac{1}{f'_{\mathrm{P}}(p_j)}\frac{\partial(p_{j+1} f'_{\mathrm{P}}(p_{j+1}))}{\partial p_{j+1}} + x_j\sqrt{\psi_j}\left(\frac{\partial(p_j f'_{\mathrm{P}}(p_j))}{\partial p_j}\right)\right].
\end{aligned} \tag{SI.144}$$

From Eqs. (SI.124), (SI.126) and (SI.144), one then has that

$$\begin{aligned}
\frac{\partial \psi_j}{\partial \theta_j} &> 0, \qquad f_{\mathrm{P}} \in \mathscr{F}_+^p \text{ or } f_{\mathrm{P}} \in \mathscr{F}_-^p \text{ with } p_j, p_{j+1} < b \\
\frac{\partial \psi_j}{\partial \theta_j} &< 0, \qquad f_{\mathrm{P}} \in \mathscr{F}_-^p \text{ with } p_j, p_{j+1} > b.
\end{aligned} \tag{SI.145}$$

Differentiating ψ_j with respect to the radius, one gets

$$\begin{aligned}
\frac{\partial \psi_j}{\partial \varrho_j} &= \frac{\partial p_{j+1}}{\partial \varrho_j}\frac{\partial \psi_j}{\partial p_{j+1}} + \frac{\partial p_j}{\partial \varrho_j}\frac{\partial \psi_j}{\partial p_j} \\
&= \frac{1}{\varrho_j}\left(p_{j+1}\frac{\partial \psi_j}{\partial p_{j+1}} + p_j\frac{\partial \psi_j}{\partial p_j}\right) \\
&= \frac{2\sqrt{\psi_j}}{\sqrt{p_j^2 + p_{j+1}^2}}\left[\frac{p_{j+1}}{p_j f'_{\mathrm{P}}(p_j)}\frac{\partial(p_{j+1} f'_{\mathrm{P}}(p_{j+1}))}{\partial p_{j+1}} + p_j p_{j+1} f'_{\mathrm{P}}(p_{j+1})\frac{\partial}{\partial p_j}\frac{1}{(p_j f'_{\mathrm{P}}(p_j))}\right] \\
&= \frac{2\psi_j}{\sqrt{p_j^2 + p_{j+1}^2}}\left[\frac{1}{f'_{\mathrm{P}}(p_{j+1})}\frac{\partial(p_{j+1} f'_{\mathrm{P}}(p_{j+1}))}{\partial p_{j+1}} - \frac{1}{f'_{\mathrm{P}}(p_j)}\frac{\partial(p_j f'_{\mathrm{P}}(p_j))}{\partial p_j}\right]
\end{aligned} \tag{SI.146}$$

where we note that the quantity between brackets in Eq. (SI.146) vanishes for $p_{j+1} = p_j$, corresponding to $\theta_j = \frac{\pi}{4}$,

$$\left.\frac{\partial \psi_j}{\partial \varrho_j}\right|_{\theta_j = \frac{\pi}{4}} = 0. \tag{SI.147}$$

The problem of establishing dynamical properties of the transformed system can now be tackled. To this aim, the strategy followed here is to partition the system into two subsystems. The first subsystem is composed of survival at pre-reproductive ages, while the second subsystem is composed of survival at reproductive ages.

3.5.4 Stability of survival to pre-reproductive ages

Let us restrict attention to the transformed subsystem $x_0, x_1, \dots, x_{\alpha-2}$, which is finite dimensional as it only includes survival at pre-reproductive ages. Define the vector $\mathbf{x}_0 = (x_0, x_1, \dots, x_{\alpha-2})$. From Eqs. (SI.21c), (SI.138) and (SI.143), this subsystem only has one equilibrium

$$x_0^* = x_1^* = \cdots = x_{\alpha-2}^* = 1, \tag{SI.148}$$

which is coherent with $p_0^* = p_1^* = \cdots = p_{\alpha-1}$ as expected from Eqs. (SI.120) and (SI.134). Moreover, by Eq. (SI.143), we have that

$$\psi_0^* = \psi_1^* = \cdots = \psi_{\alpha-2}^* = 1. \tag{SI.149}$$

The dynamical behaviour induced on this subsystem by the functions found in the two sets defined in Section 3.3 is analysed separately for each set. For both cases, we can use Eqs. (SI.21) and (SI.138) to define the map

$$S_j((x_n(t))) = x_j(t+1) = x_j(t) + \delta c_j^p(t)\left[1 - \psi_j(t)\right], \quad j = 0, 1, \dots, \alpha-2, \tag{SI.150}$$

which takes as argument the sequence (x_n) at t and returns the term $x_j(t+1)$ with $j = 0, 1, \dots, \alpha-2$ of the sequence (x_n) at time $t+1$. It is also useful to define

$$\Delta^* x_j = x_j - x_j^* = x_j - 1, \quad j = 0, 1, \dots, \alpha-2. \tag{SI.151}$$

which gives the difference between x_j, $j = 0, 1, \dots, \alpha-2$, and its equilibrium value, and to let

$$d(\mathbf{x}_0, \mathbf{x}_0^*) = \sqrt{\sum_{i=0}^{\alpha-2} (\Delta^* x_j)^2} \geq 0, \tag{SI.152}$$

be the Euclidean distance between $\mathbf{x}_0$ and its equilibrium value.

Case 1: $f_{\mathrm{P}} \in \mathscr{F}_+^p$.

Suppose that, at some time t, the subsystem is not at equilibrium. Then $x_j(t) \neq 1$ for some $j = 0, ..., \alpha-2$. If $x_j(t) = 1$, then $x_j(t+1) = x_j(t)$ by Eqs. (SI.143) and (SI.150). If $x_j(t) > 1$, then $x_j(t+1) < x_j(t)$ by Eqs. (SI.141) and (SI.150). If $x_j(t) < 1$, then $x_j(t+1) > x_j(t)$ again by Eqs. (SI.141) and (SI.150). Therefore, the quantity x_j, if it is equal to 1, it will remain equal to 1, while if it is different from 1, it always tends to move towards the value 1, i.e. if it is above this value it will decrease, while if it is below this value it will increase. As selection is assumed weak ($0 < \delta \ll 1$), steps towards 1 will always be very small and each x_j, $j = 0, ..., \alpha-2$ should eventually end up in some relatively small neighbourhood of its equilibrium $x_j^* = 1$, $j = 0, ..., \alpha-2$. To check stability at this equilibrium, we can differentiate the map in Eq. (SI.150) as follows

$$\frac{\partial S_j}{\partial x_i} = \delta_{i,j} + \delta \frac{\partial c_j^p}{\partial x_i}(1 - \psi_j) - \delta c_j^p \frac{\partial \psi_j}{\partial x_i}, \qquad j = 0, 1, \dots, \alpha-2, \quad i = 0, 1, \dots \tag{SI.153}$$

where $\delta_{i,j}$ is Kronecker delta (i.e. $\delta_{i,j} = 1$ when $i = j$ and $\delta_{i,j} = 0$ when $i \neq j$). We note that it is sufficient that the subsystem $\mathbf{x}_0$ is at equilibrium that the second term on the right hand side of Eq. (SI.153) vanishes because of Eq. (SI.149). Using polar coordinates, we then look at the derivative in the third term on the right hand side of Eq. (SI.153),

$$\frac{\partial \psi_j(\theta_j, \varrho_j)}{\partial x_i} = \frac{\partial \psi_j}{\partial \theta_j}\frac{\partial \theta_j}{\partial x_i} + \frac{\partial \psi_j}{\partial \varrho_j}\frac{\partial \varrho_j}{\partial x_i}, \qquad j = 0, \dots, \alpha-2, \quad i = 0, 1, \dots \tag{SI.154}$$

Since $\theta_j = \arctan x_j$,

$$\frac{\partial \psi_j}{\partial \theta_j}\frac{\partial \theta_j}{\partial x_i} = \frac{\partial \psi_j}{\partial \theta_j}\frac{\delta_{i,j}}{1 + x_i^2} \tag{SI.155}$$

The subsystem equilibrium in Eq. (SI.148) implies $\theta_0^* = \cdots = \theta_{\alpha-2}^* = \frac{\pi}{4}$. By Eq. (SI.147), this in turn implies that

$$\left(\frac{\partial \psi_j}{\partial \varrho_j}\frac{\partial \varrho_j}{\partial x_i}\right)\bigg|_{\mathbf{x}_0=\mathbf{x}_0^*} = 0, \qquad j = 0, \ldots, \alpha-2, \quad i = 0, 1, \ldots \tag{SI.156}$$

Therefore,

$$\frac{\partial \psi_j}{\partial x_i}\bigg|_{\mathbf{x}_0=\mathbf{x}_0^*} = \frac{\delta_{i,j}}{1+(x_i^*)^2}\frac{\partial \psi_j}{\partial \theta_j}\bigg|_{\mathbf{x}_0=\mathbf{x}_0^*}, \qquad j = 0, \ldots, \alpha-2, \quad i = 0, 1, \ldots \tag{SI.157}$$

Suppose there exists a (sufficiently small) Euclidean distance M from the equilibrium $\mathbf{x}_0^*$, a vector with $\alpha - 1$ components each equal to 1, see Eq. (SI.148), so that the following dynamics

$$\Delta^* x_j(t+1) \approx \Delta^* x_j(t)\frac{\partial S_j}{\partial x_j}\bigg|_{\mathbf{x}_0=\mathbf{x}_0^*}, \quad j = 0, 1, \ldots, \alpha-2 \tag{SI.158}$$

for the quantity in Eq. (SI.151) hold. The equation in (SI.158) linearly approximates the dynamics of the deviations of the subsystem from its equilibrium so long as $\mathbf{x}_0(t)$ is within Euclidean distance M from $\mathbf{x}_0^*$, i.e. so long as

$$d(\mathbf{x}_0(t), \mathbf{x}_0^*) \leq M. \tag{SI.159}$$

Taking absolute values of Eq. (SI.158) and using Eqs. (SI.149), (SI.153) and (SI.157),

$$|\Delta^* x_j(t+1)| \approx |\Delta^* x_j(t)| \left(1 - \delta \frac{c_j^p(t)}{1+(x_j^*)^2}\frac{\partial \psi_j}{\partial \theta_j}\bigg|_{\mathbf{x}_0=\mathbf{x}_0^*}\right), \quad j = 0, 1, \ldots, \alpha-2, \tag{SI.160}$$

In this equation, the quantity between parentheses in Eq. (SI.160) satisfies the inequalities

$$0 < 1 - \delta \frac{c_j^p(t)}{1+(x_j^*)^2}\frac{\partial \psi_j}{\partial \theta_j}\bigg|_{\mathbf{x}_0=\mathbf{x}_0^*} < 1, \quad j = 0, 1, \ldots, \alpha-2, \tag{SI.161}$$

in virtue of Eq. (SI.145) and the assumption of sufficiently weak selection ($0 < \delta \ll 1$). In this quantity, $c_j^p(t)$ is not at equilibrium because, as Eq. (SI.139) shows, it is a function of the whole sequence (x_n) and not only of $\mathbf{x}_0$.

Hence, Eq. (SI.160) shows that

$$\begin{aligned} &\text{If } |\Delta^* x_j(t)| = 0, \text{ then } |\Delta^* x_j(t+1)| = 0, && j = 0, 1, \ldots, \alpha-2, \\ &\text{If } |\Delta^* x_j(t)| > 0, \text{ then } |\Delta^* x_j(t+1)| < |\Delta^* x_j(t)|, && j = 0, 1, \ldots, \alpha-2. \end{aligned} \tag{SI.162}$$

As a consequence, when $\mathbf{x}_0(t)$ is within distance M from $\mathbf{x}_0^*$, the components of $\mathbf{x}_0(t)$ that do not deviate from their equilibrium values remain at equilibrium values in one time step, while all nonzero deviations shrink in absolute value in one time step. Since $|z|^2 = z^2$ and squaring preserves order, we can use Eqs. (SI.152), (SI.159) and (SI.162) to compare Euclidean distances, if $0 < d(\mathbf{x}_0(t), \mathbf{x}_0^*) \leq M$, then

$$d(\mathbf{x}_0(t+1), \mathbf{x}_0^*) = \sqrt{\sum_{i=0}^{\alpha-2}(\Delta^* x_j(t+1))^2} < \sqrt{\sum_{i=0}^{\alpha-2}(\Delta^* x_j(t))^2} = d(\mathbf{x}_0(t), \mathbf{x}_0^*) \leq M. \tag{SI.163}$$

and if $d(\mathbf{x}_0(t), \mathbf{x}_0^*) = 0$, then $d(\mathbf{x}_0(t+1), \mathbf{x}_0^*) = 0$. Therefore, if $\mathbf{x}_0(t)$ is within positive distance M from $\mathbf{x}_0^*$, then $\mathbf{x}_0(t+1)$ also is within distance M from $\mathbf{x}_0^*$ and the dynamics in Eq. (SI.158)

apply again. Since $d(\mathbf{x}_0, \mathbf{x}_0^*)$ is bounded below by zero and $d(\mathbf{x}_0(t+1), \mathbf{x}_0^*) < d(\mathbf{x}_0(t), \mathbf{x}_0^*)$ when $d(\mathbf{x}_0(t), \mathbf{x}_0^*) \leq M$, by monotone convergence we have

$$\lim_{t\to\infty} d(\mathbf{x}_0(t), \mathbf{x}_0^*) = 0, \tag{SI.164}$$

i.e. the distance between $\mathbf{x}_0(t)$ and $\mathbf{x}_0^*$ eventually goes to zero so long as the former is found within a sufficiently small neighbourhood of the latter that the linearized dynamics in Eq. (SI.158) hold. Hence, when $f_\mathrm{P} \in \mathscr{F}_+^p$, the equilibrium in Eq. (SI.148) is stable.

For the subcase $f_\mathrm{P}(p) = p^q$ with $q > 0$, a stronger result about the stability of the subsystem $\mathbf{x}_0$ is obtained by using an approach based on Lyapunov method. From Eqs. (SI.134) and (SI.140), we can write

$$\psi_j = x_j^{2q}, \qquad \text{with } f_\mathrm{P}(p) = p^q \text{ with } q > 0, \tag{SI.165}$$

which shows that ψ_j only depends on x_j and $\psi_j'(x_j) = 2qx_j^{2q-1}$. Define

$$V^p = \sum_{j=0}^{\alpha-2}(1-\psi_j)^2 = \sum_{j=0}^{\alpha-2}(1-x_j^{2q})^2, \qquad \text{with } f_\mathrm{P}(p) = p^q \text{ with } q > 0. \tag{SI.166}$$

where, because of Eq. (SI.21c), we have that $1 - x_j^{2q}$ corresponds to the bracketed quantity in Eq. (SI.138) for $j = 0, 1, \ldots, \alpha-2$. Clearly, V^p is always positive except at the subsystem equilibrium in Eqs. (SI.148) and (SI.149), where it vanishes. Therefore, V^p is a candidate Lyapunov function.

Suppose $\mathbf{x}_0$ is not at equilibrium at t so that $V^p(t) > 0$. Hence, $(1-x_j^{2q}(t))^2 > 0$ for at least some $j = 0, 1 \ldots, \alpha-2$. Let $K \subset \{0, 1 \ldots, \alpha-2\}$ be the non-empty index set such that $j \in K$ if and only if $(1-x_j^{2q}(t))^2 > 0$. Note that if $(1-x_j^{2q}(t))^2 = 0$ for some $j \notin K$, then, by Eq. (SI.150) $x_j(t+1) = x_j(t)$ and $x_j^{2q}(t+1) = x_j^{2q}(t)$ and, therefore, $(1-x_i^{2q}(t+1))^2 = 0$. Thus, for any term that is equal to zero in the sum given by $V^p(t)$, the corresponding term in the sum given by $V^p(t+1)$ is still equal to zero. Consider then terms in the sum given by $V^p(t)$ with index in K. Neglecting terms of order δ^2 and using Eq. (SI.150) to approximate both $x_j^{2q}(t+1)$ and $x_j^{4q}(t+1)$ with a first order Taylor expansion around $\delta = 0$ and to get an expression for $\Delta x_j(t) := x_j(t+1) - x_j(t)$, $j = 0, 1 \ldots, \alpha-2$, we have that

$$\begin{aligned}(1-x_j^{2q}(t+1))^2 - (1-x_j^{2q}(t))^2 &\approx -4qx_j^{2q-1}(t)\Delta x_j(t) + 4x_j^{2q}(t)qx_j^{2q-1}(t)\Delta x_j(t) \\ &= -4q\delta c_j^p(t)(1-x_j^{2q}(t))^2 x_j^{2q-1}(t) \\ &< 0, \qquad j \in K\end{aligned} \tag{SI.167}$$

Therefore,

$$\text{if } V^p(t) > 0, \text{ then } V^p(t+1) - V^p(t) < 0, \quad \text{with } f_\mathrm{P}(p) = p^q \text{ with } q > 0, \tag{SI.168}$$

and V^p is a strict Lyapunov function for the subsystem: flat pre-reproductive survival is a globally stable equilibrium when f_P is a power function.

As only a subsystem of the transformed system is here considered, it is not possible to get back to the original coordinates. However, it can be concluded that survival at pre-reproductive ages is globally attracted to the same equilibrium value irrespective of age, but the analysis leaves this value undetermined.

Case 2: $f_\mathrm{P} \in \mathscr{F}_-^p$.
Here we consider two subcases. First, suppose that b is only slightly smaller than 1 so that $p_j^* < b$, $j = 0, \ldots, \alpha-1$, if an equilibrium exists, and $p_j(t) < b$, $j = 0, \ldots, \alpha-1$ for all t. Then, the same

arguments as before apply and flat age-specific survival at pre-reproductive ages is an attracting, stable equilibrium.

Second, suppose instead that, if an equilibrium of survival at pre-reproductive ages exists, $p_j^* > b$, $j = 0, \ldots, \alpha - 1$, e.g. because $0 < b \ll 1$. Since $p_j^* > b$, $j = 0, \ldots, \alpha - 1$ and weak selection ($0 < \delta \ll 1$) imposes small changes per time step, it is necessary that the dynamics of pre-reproductive survival $p_j(t)$, $j = 0, ..., \alpha - 1$ eventually remain in the interval

$$L_j = (p_j^* - \min\{1 - p_j^*, p_j^* - b\}, p_j^* + \min\{1 - p_j^*, p_j^* - b\}), \quad j = 0, \ldots, \alpha - 1, \tag{SI.169}$$

to have a chance of getting to equilibrium. We shall then study the dynamics of pre-reproductive survival assuming that these unfold so that $p_j(t) \in L_j$, $j = 0, \ldots, \alpha - 1$, for all t. As a consequence, $p_j(t) > b$, $j = 0, \ldots, \alpha - 1$, for all t. Suppose that, at some time t, pre-reproductive survival is not at equilibrium so that $p_j(t) \neq p_j^*$ for some $j = 0, \ldots, \alpha - 1$. In the transformed subsystem $\mathbf{x}_0(t)$, this means that $x_j(t) \neq 1$ for some $j = 0, \ldots, \alpha - 2$. Let then

$$d(\mathbf{x}_0(t), \mathbf{x}_0^*) = \sqrt{\sum_{i=0}^{\alpha-2} (x_j(t) - 1)^2} > 0 \tag{SI.170}$$

be the Euclidean distance between $\mathbf{x}_0(t)$ and the equilibrium $\mathbf{x}_0^*$, which is a vector with $\alpha - 1$ components all equal to 1, see Eq. (SI.148). If $x_j(t) = 1$, then $x_j(t+1) = x_j(t)$ by Eqs. (SI.143) and (SI.150). If $x_j(t) > 1$, then $x_j(t+1) > x_j(t) > 1$ by Eqs. (SI.142) and (SI.150). If $x_j(t) < 1$, then $x_j(t+1) < x_j(t) < 1$ again by Eqs. (SI.142) and (SI.150). Hence, we obtain the following relationship about the absolute values of the deviations,

$$\begin{aligned} &\text{If } |\Delta^* x_j(t)| = 0, \text{ then } |\Delta^* x_j(t+1)| = 0, && j = 0, 1, \ldots, \alpha - 2 \\ &\text{If } |\Delta^* x_j(t)| > 0, \text{ then } |\Delta^* x_j(t+1)| > |\Delta^* x_j(t)|, && j = 0, 1, \ldots, \alpha - 2. \end{aligned} \tag{SI.171}$$

Since $|z|^2 = z^2$ and squaring preserves order, we can use Eqs. (SI.152), (SI.170) and (SI.171) to compare Euclidean distances,

$$d(\mathbf{x}_0(t+1), \mathbf{x}_0^*) = \sqrt{\sum_{i=0}^{\alpha-2} (\Delta^* x_j(t+1))^2} > \sqrt{\sum_{i=0}^{\alpha-2} (\Delta^* x_j(t))^2} = d(\mathbf{x}_0(t), \mathbf{x}_0^*) > 0. \tag{SI.172}$$

Therefore, when $\mathbf{x}_0(t)$ is not at equilibrium, the distance from this will increase. The equilibrium is then unstable.

As instability of the subsystem $x_0, x_1, \ldots, x_{\alpha-2}$ implies instability of the whole system, negative ageing cannot be a stable equilibrium if $p_j^* > b$ for $j = 0, ..., \alpha - 1$.

3.5.5 Set stability of survival at reproductive ages

Let us restrict attention to the subsequence $(x_n)_{\alpha-1} = x_{\alpha-1}, x_\alpha, \ldots$, which only includes survival into the first reproductive age and subsequent survival. The aim here is to characterize regions of the state space that attract or repel the dynamics of $(x_n)_{\alpha-1}$. Define the sets

$$\begin{aligned} U^p &:= \{(x_n)_{\alpha-1} : x_j > 0, && j = \alpha - 1, \alpha, \ldots\} \\ A_\leq^p &:= \{(x_n)_{\alpha-1} : 0 < x_j \leq 1, && j = \alpha - 1, \alpha, \ldots\} \\ A_<^p &:= \{(x_n)_{\alpha-1} : 0 < x_j < 1, && j = \alpha - 1, \alpha, \ldots\} \\ A_>^p &:= \{(x_n)_{\alpha-1} : x_j > 1, && j = \alpha - 1, \alpha, \ldots\} \end{aligned}. \tag{SI.173}$$

The set U^p is the entire state space where the dynamics of $(x_n)_{\alpha-1}$ unfold. The possibility that $x_j = 0$ for some $j = \alpha - 1, \alpha, \ldots$ is excluded. This is because survival is assumed initially positive and the dynamics in

Eq. (SI.33) should guarantee its strict positivity throughout. In the transformed system, this should preserve the positivity of x_j, $j = 0, 1, \dots$.

Any complement of a subset of U^p should be meant as a complement relative to U^p excluding the 0 boundary. The set $A^p_{\leq}$ is the set of all life histories with adult survival that either stays constant or decreases with age. $A^p_{<}$ is the interior of $A^p_{\leq}$ and, by the definitions in Section 1.2, it is the set of all life histories with ageing in survival. $A^p_{>}$ is the set of all life histories with negative ageing in survival. The dynamical behaviour induced by the two sets of functions is analysed separately.

Case 1: $f_{\mathrm{P}} \in \mathscr{F}^p_{+}$.

As shown in Section 3.4.1, at equilibrium, $p^*_{j+1} < p^*_j$, $j = \alpha - 1, \alpha, \dots$. Therefore, $x^*_j < 1$, $j = \alpha - 1, \alpha, \dots$ and $(x^*_n)_{\alpha-1} \in A^p_{<} \subset A^p_{\leq}$. Here three properties of $A^p_{\leq}$ are shown under the dynamics in Eq. (SI.138) assuming sufficiently weak selection:

(i) $A^p_{\leq}$ is an invariant set: if $(x_n(t))_{\alpha-1} \in A^p_{\leq}$, then $(x_n(t+1))_{\alpha-1} \in A^p_{\leq}$. To show this, observe that the quantities x_j change by very small amounts at each time step because of weak selection. Hence, the passage from $x_j(t) \leq 1$ to $x_j(t+1) > 1$ for some $j = \alpha - 1, \alpha, \dots$ can only happen in some small neighbourhood around the boundary between $A^p_{\leq}$ and its complement. In this neighbourhood, $x_j \approx 1$. Therefore, $p_j \approx p_{j+1}$ and $\theta_j \approx \frac{\pi}{4}$. By (SI.147), the derivative $\frac{\partial \psi_j}{\partial \varrho_j}$ tends to vanish in this neighbourhood. Noting, as in Eqs. (SI.154)-(SI.157), that $\theta_j = \arctan(x_j)$ only is a function of x_j we can write the total differential

$$\mathrm{d}\psi_j \approx \frac{\partial \psi_j}{\partial x_j} \Delta x_j = \frac{\partial \theta_j}{\partial x_j} \frac{\partial \psi_j}{\partial \theta_j} \Delta x_j = \frac{1}{1 + x_j^2} \frac{\partial \psi_j}{\partial \theta_j} \Delta x_j \qquad \text{(SI.174)}$$

only in terms of the change in x_j and the sensitivity of ψ_j to it, because the x_i's with $i \neq j$ have an influence on ψ_j only via ϱ_j. This differential is meant to be valid when $x_j \approx 1$. We also note that, in this differential, the quantity $\frac{\partial \psi_j}{\partial \theta_j}$ is positive by Eq. (SI.145).

Suppose now, towards a contradiction, that $(x_n(t))_{\alpha-1} \in A^p_{\leq}$ and $(x_n(t+1))_{\alpha-1} \notin A^p_{\leq}$. Then, by Eq. (SI.173), $x_j(t) \leq 1$ for all $j = \alpha - 1, \alpha, \dots$, while $x_j(t+1) > 1$ for at least some $j = \alpha - 1, \alpha, \dots$. Let $K \subseteq \{\alpha - 1, \alpha, \dots\}$ be the index set such that $j \in K$ if and only of $x_j(t+1) > 1$. Then, $x_j(t+1) > 1 \geq x_j(t)$ for $j \in K$. This implies, by Eq. (SI.138), that $\frac{H_{j+1}(t)}{H_j(t)} - \psi_j(t) > 0$ for $j \in K$ and, by Eq. (SI.141), that $\psi_j(t+1) > 1$ for $j \in K$. By Eq. (SI.21d), $\frac{H_{j+1}(t)}{H_j(t)} < 1$, $j = \alpha - 1, \alpha, \dots$, independently of t. Hence, using the total differential $\mathrm{d}\psi_j(t)$ in Eq. (SI.174) as a substitute for $\Delta\psi_j(t) = \psi_j(t+1) - \psi_j(t)$,

$$\frac{H_{j+1}(t)}{H_j(t)} < \psi_j(t+1) \approx \psi_j(t) + \frac{\Delta x_j(t)}{1 + x_j^2(t)} \frac{\partial \psi_j}{\partial \theta_j}(t), \quad j \in K. \qquad \text{(SI.175)}$$

Subtracting $\psi_j(t)$ from both sides of this inequality and then dividing by the positive quantity $\frac{H_{j+1}(t)}{H_j(t)} - \psi_j(t)$ and using Eq. (SI.138),

$$1 < \delta \frac{c^p_j(t)}{1 + x_j^2(t)} \frac{\partial \psi_j}{\partial \theta_j}(t), \quad j \in K. \qquad \text{(SI.176)}$$

But this contradicts the assumption of weak selection (i.e. $0 < \delta \ll 1$). Therefore, it is not the case that $(x_n(t))_{\alpha-1} \in A^p_{\leq}$ and $(x_n(t+1))_{\alpha-1} \notin A^p_{\leq}$. Equivalently, if $(x_n(t))_{\alpha-1} \in A^p_{\leq}$, then $(x_n(t+1))_{\alpha-1} \in A^p_{\leq}$.

(ii) $A^p_{\leq}$ pushes trajectories into its interior: if $(x_n(t))_{\alpha-1} \in A^p_{\leq}$, then $(x_n(t+1))_{\alpha-1} \in A^p_{<}$. To show this, suppose $(x_n(t))_{\alpha-1} \in A^p_{\leq}$. If $x_j(t) = 1$ for some $j = \alpha - 1, \alpha, \dots$, then, by Eq. (SI.143), $\psi_j(t) = 1$. By Eq. (SI.21d), $\frac{H_{j+1}(t)}{H_j(t)} < 1$, $j = \alpha - 1, \alpha, \dots$, independently of

t. Hence, $\frac{H_{j+1}(t)}{H_j(t)} - \psi_j(t) < 0$ and, by Eq. (SI.138), $x_j(t+1) < x_j(t) = 1$. If $x_j(t) < 1$ for some $j = \alpha - 1, \alpha, \ldots$, then the supposition that $\psi_j(t+1) = 1$ along with the assumption of weak selection leads to the same contradiction as in Eq. (SI.176) via an argument analogous to that used to prove invariance of $A^p_{\leq}$. Thus, if $x_j(t) < 1$, then $x_j(t+1) < 1$.

Therefore, if $x_j(t) \leq 1$, $j = \alpha - 1, \alpha, \ldots$, then $x_j(t+1) < 1$, $j = \alpha - 1, \alpha, \ldots$.

(iii) $A^p_{\leq}$ is a stable attracting set. Trajectories from outside of $A^p_{\leq}$ end up in it and stay there. To show this, we combine Eqs. (SI.7) and (SI.10) with the fact that the sequence $1, 1, \ldots$ is the boundary of $A^p_{\leq}$ to get the distance of the sequence $(x_n)_{\alpha-1}$ from the set $A^p_{\leq}$ as

$$d_{\alpha-1}((x_n)_{\alpha-1}, A^p_{\leq}) = \sum_{i=\alpha-1}^{\infty} \frac{\Theta(x_i - 1)}{2^i} \frac{|x_i - 1|}{1 + |x_i - 1|} \tag{SI.177}$$

where Θ is the unit step function, i.e. $\Theta(z) = 1$ for $z \geq 0$ and $\Theta(z) = 0$ for $z < 0$. The unit step function takes care of the fact that if any term of $(x_n)_{\alpha-1}$ is smaller than 1, then the contribution of the corresponding term in the series in Eq. (SI.177) must be zero, because there is some sequence in $A^p_{\leq}$ that has a term that has both the same index and the same value. Suppose $(x_n(t))_{\alpha-1} \notin A^p_{\leq}$ so that $d_{\alpha-1}((x_n(t))_{\alpha-1}, A^p_{\leq}) > 0$. Then, $x_j(t) > 1$ for some $j = \alpha - 1, \alpha, \ldots$ Let $K \subseteq \{\alpha - 1, \alpha, \ldots\}$ be the index set such that $j \in K$ if and only if $x_j(t) > 1$, i.e. K exclusively contains the indexes of the positive terms in the series given by $d_{\alpha-1}((x_n(t))_{\alpha-1}, A^p_{\leq})$, while all other terms are equal to zero. Let us first consider those terms that are equal to zero in this series. By invariance of $A^p_{\leq}$, if $x_j(t) \leq 1$ for $j \notin K$, then $x_j(t+1) \leq 1$ for $j \notin K$. Thus, for any term that is equal to zero in the series given by $d_{\alpha-1}((x_n(t))_{\alpha-1}, A^p_{\leq})$, the corresponding term in the series given by $d_{\alpha-1}((x_n(t+1))_{\alpha-1}, A^p_{\leq})$ is still equal to zero. Consider then terms that are greater than zero in the series given by $d_{\alpha-1}((x_n(t))_{\alpha-1}, A^p_{\leq})$. By Eq. (SI.141), $\psi_j(t) > 1$ for $j \in K$. By Eq. (SI.21d), $\frac{H_{j+1}(t)}{H_j(t)} < 1$, $j = \alpha - 1, \alpha, \ldots$, independently of t. By Eq. (SI.138), $x_j(t+1) < x_j(t)$ for $j \in K$. Recall that $x_j(t) - 1 > 0$ for $j \in K$. If $x_j(t+1) - 1 \leq 0$, then

$$\begin{aligned} \frac{\Theta(x_j(t+1) - 1)|x_j(t+1) - 1|}{1 + |x_j(t+1) - 1|} &= 0 \\ &< \frac{x_j(t) - 1}{1 + x_j(t) - 1} \\ &= \frac{\Theta(x_j(t) - 1)|x_j(t) - 1|}{1 + |x_j(t) - 1|}, \qquad j \in K. \end{aligned} \tag{SI.178}$$

If $x_j(t+1) - 1 > 0$, then, using the fact that $z/(1+z)$ is strictly increasing on $(-1/2, \infty)$ and, therefore, preserves order on this interval,

$$\begin{aligned} \frac{\Theta(x_j(t+1) - 1)|x_j(t+1) - 1|}{1 + |x_j(t+1) - 1|} &= \frac{x_j(t+1) - 1}{1 + x_j(t+1) - 1} \\ &< \frac{x_j(t) - 1}{1 + x_j(t) - 1} \\ &= \frac{\Theta(x_j(t) - 1)|x_j(t) - 1|}{1 + |x_j(t) - 1|}, \qquad j \in K. \end{aligned} \tag{SI.179}$$

Either way, all positive terms in the series given by $d_{\alpha-1}((x_n(t))_{\alpha-1}, A^p_{\leq})$ are larger than the corresponding terms in the series given by $d_{\alpha-1}((x_n(t+1))_{\alpha-1}, A^p_{\leq})$. Hence, $d_{\alpha-1}((x_n(t+1))_{\alpha-1}, A^p_{\leq}) < d_{\alpha-1}((x_n(t))_{\alpha-1}, A^p_{\leq})$. By monotone convergence, we then have that

$$\lim_{t\to\infty} d_{\alpha-1}((x_n(t))_{\alpha-1}, A^p_{\leq}) = 0 \tag{SI.180}$$

for any $(x_n(t))_{\alpha-1} \in U^p$.

Hence, when $f_{\mathrm{P}} \in \mathscr{F}_{+}^{p}$, the set $A_{\leq}^{p}$ fulfils the properties [16] to be a globally stable attracting set of the dynamics of $(x_n)_{\alpha-1}$. In virtue of property (ii), all trajectories lead to $A_{<}^{p}$ so that the distance between this set and $(x_n)_{\alpha-1}$ also goes to zero. This distance is also measured via Eq. (SI.177) because the distance between an open set and a point on its boundary is zero. Eventually, ageing in survival evolves from any initial life history.

Case 2: $f_{\mathrm{P}} \in \mathscr{F}_{-}^{p}$.

Two subcases are considered.

First, suppose b is only slightly smaller than 1 and the evolving sequence $(p_n(t))$ is bounded above by b for all t. In this case, the same arguments apply as for the Case 1 ($f \in \mathscr{F}_{+}^{p}$) and ageing in survival is expected to evolve.

Second, let $0 < b \ll 1$ and assume that the evolving sequence $(p_n(t))$ is bounded below by b for all t. As shown in Section 3.4.2, at equilibrium, $p_{j+1}^{*} > p_j^{*}$, $j = \alpha-1, \alpha, \ldots$. Therefore, $x_j^{*} > 1$, $j = \alpha-1, \alpha, \ldots$ and $(x_n^{*})_{\alpha-1} \in A_{>}^{p}$. Here, it is shown that the set $A_{>}^{p}$, which contains any equilibrium of $(x_n^{*})_{\alpha-1}$ when $f_{\mathrm{P}} \in \mathscr{F}_{-}^{p}$, is a repelling set under the dynamics in Eq. (SI.138). Combining Eqs. (SI.7) and (SI.10), the distance between $(x_n)_{\alpha-1}$ and $A_{>}^{p}$ is

$$d_{\alpha-1}((x_n)_{\alpha-1}, A_{>}^{p}) = \sum_{i=\alpha-1}^{\infty} \frac{\Theta(-(x_i-1))}{2^i} \frac{|x_i-1|}{1+|x_i-1|} \tag{SI.181}$$

where the unit step function Θ takes care of the fact that if any term of $(x_n)_{\alpha-1}$ is greater than 1, then the contribution of the corresponding term in the series in Eq. (SI.181) must be zero, because there is some sequence in $A_{>}^{p}$ that has a term that has both the same index and the same value. We also note that if $y_j = 1$ for $j = \alpha, \alpha+1, \ldots$, then the distance in Eq. (SI.181) is zero, as it should be, since the distance between a point on the boundary of an open set and this set is zero. Suppose $(x_n(t))_{\alpha-1} \notin A_{>}^{p}$. Then, by Eq. (SI.173), $x_j(t) \leq 1$ for some $j = \alpha-1, \alpha, \ldots$, and

$$d_{\alpha-1}((x_n(t))_{\alpha-1}, A_{>}^{p}) > 0. \tag{SI.182}$$

Let $K \subseteq \{\alpha-1, \alpha, \ldots\}$ be the index set such that $j \in K$ if and only if $x_j(t) \leq 1$. By Eqs. (SI.142)-(SI.143), $\psi_j(t) \geq 1$ for $j \in K$. By Eq. (SI.21d), $\frac{H_{j+1}(t)}{H_j(t)} < 1$, $j = \alpha-1, \alpha, \ldots$, independently of t. Thus, $\frac{H_{j+1}(t)}{H_j(t)} - \psi_j(t) < 0$ and, by Eq. (SI.138), $x_j(t+1) < x_j(t)$ for $j \in K$. Therefore, $x_j(t+1) < x_j(t) \leq 1$ for $j \in K$. Using the fact that $z/(1+z)$ is strictly increasing on $(-1/2, \infty)$ and, therefore, preserves order on this interval,

$$\begin{aligned} \frac{\Theta(-(x_j(t+1)-1))|x_j(t+1)-1|}{1+|x_j(t+1)-1|} &= \frac{|x_j(t+1)-1|}{1+|x_j(t+1)-1|} \\ &= \frac{1-x_j(t+1)}{1+1-x_j(t+1)} \\ &> \frac{1-x_j(t)}{1+1-x_j(t)} \\ &= \frac{\Theta(-(x_j(t)-1))|x_j(t)-1|}{1+|x_j(t)-1|}, \qquad j \in K. \end{aligned} \tag{SI.183}$$

Therefore, all positive terms in the series given by $d_{\alpha-1}((x_n(t+1))_{\alpha-1}, A_{>}^{p})$ are larger than the corresponding terms in the series given by $d_{\alpha-1}((x_n(t))_{\alpha-1}, A_{>}^{p})$. As for the zero terms in the series given by $d_{\alpha-1}((x_n(t))_{\alpha-1}, A_{>}^{p})$, the corresponding terms in the series given by $d_{\alpha-1}((x_n(t+1))_{\alpha-1}, A_{>}^{p})$ are either zero or positive. Either way, we have that

$$d_{\alpha-1}((x_n(t+1))_{\alpha-1}, A_{>}^{p}) > d_{\alpha-1}((x_n(t))_{\alpha-1}, A_{>}^{p}) > 0. \tag{SI.184}$$

Hence, if $(x_n(t))_{\alpha-1} \notin A^p_>$, then $(x_n(t+1))_{\alpha-1} \notin A^p_>$ and the distance between $(x_n(t))_{\alpha-1}$ and the set $A^p_>$, where an equilibrium (if any exists) is found, keeps increasing with time. The set of life histories with negative ageing in survival $A^p_>$ is repelling. Negative ageing in survival can not evolve from an initial life history without negative ageing.

3.6 Minimal model

In the previous section, equilibrium states of survival were not characterized in terms of their number and stability. Here we obtain such a characterization under the simplifying assumption that there are only two age classes, i.e. that $p_2(t) = 0$ for all t, and reproduction starts from the first ($\alpha = 1$), i.e. $m_1, m_2 > 0$. This model is inspired by that in [17]. In this case, the Euler-Lotka equation (SI.2) for a demographically stationary population ($\lambda = 1$) is

$$p_0 m_1 + p_0 p_1 m_2 = 1. \tag{SI.185}$$

Since $p_0, p_1 \leq 1$, we have that $p_0 m_1 + p_0 p_1 m_2 \leq m_1 + m_2$. If $m_1 + m_2 < 1$, Eq. (SI.185) cannot be satisfied. Therefore, it is required that $m_1 + m_2 \geq 1$. Setting p_1 equal to p_0 (i.e. neither ageing nor negative ageing) in Eq. (SI.185), the left hand side becomes a second degree polynomial $\zeta(p_0) = p_0 m_1 + p_0^2 m_2$ in p_0. As $\zeta'(p_0) > 0$ for $p_0 > 0$ and $\zeta(0) = 0$, the equation $\zeta(p_0) = 1$ has a unique positive root $\hat{p}_0$,

$$\hat{p}_0 = \frac{-m_1 + \sqrt{m_1^2 + 4m_2}}{2m_2}. \tag{SI.186}$$

We want to show that this root is in the interval $(0, 1]$. We already established that $\hat{p}_0$ is a positive root. We need only to show $\hat{p}_0 \leq 1$. Suppose, towards a contradiction, that $\hat{p}_0 > 1$. If we set the right hand side of Eq. (SI.186) greater than 1, we obtain $m_1 + m_2 < 1$. But this contradicts the requirement that $m_1 + m_2 \geq 1$. Hence, the root $\hat{p}$ of $\zeta(p_0)$ is in the interval $(0, 1]$ for the initial choice of $m_1, m_2 > 0$ and $m_1 + m_2 \geq 1$. Within this simplified model, we can assume that ageing is present when $p_0 > p_1$, while there is negative ageing when $p_0 < p_1$. The just obtained root allows to readily detect ageing or its absence in this model: there is ageing in survival whenever $\hat{p}_0 < p_0 \leq 1$, while there is negative ageing in survival whenever $0 < p_0 < \hat{p}_0$.

To understand the dynamics in this model, we let the summation in Eq. (SI.117) only go to 2 instead of infinity. Then, we take logs of both sides of the resulting equation and a first-order Taylor expansion around $\delta = 0$ to get two dynamical equations,

$$\ln p_j(t+1) \approx \ln p_j(t) + \delta \left(\frac{H_j(t)}{p_j^2(t) f'_{\mathrm{P}}(p_j(t))^2} - \sum_{i=0}^{1} \frac{H_i^2(t)}{p_i^2(t) f'_{\mathrm{P}}(p_i(t))^2} \right), \quad j = 0, 1. \tag{SI.187}$$

Using Eq. (SI.185), we can write $p_1 = \frac{1 - p_0 m_1}{p_0 m_2}$. From this and setting $\alpha = 1$ while imposing a maximum age $\omega = 2$ in Eqs. (SI.4) and (SI.20), we get $T(t) = 2 - p_0(t) m_1$, $H_0(t) = \frac{1}{T(t)}$ and $H_1(t) = \frac{1 - p_0(t) m_1}{T(t)}$. Using the expressions we just derived, we reduce Eq. (SI.187) to a single dynamical equation

$$\begin{aligned}
\ln p_0(t+1) &\approx \ln p_0(t) + \delta \left[\frac{H_0(t)}{p_0^2(t) f'_{\mathrm{P}}(p_0(t))^2} - \frac{H_0^2(t)}{p_0^2(t) f'_{\mathrm{P}}(p_0(t))^2} - \frac{H_1^2(t)}{p_1^2(t) f'_{\mathrm{P}}(p_1(t))^2} \right] \\
&= \ln p_0(t) + \frac{\delta}{T^2(t)} \left[\frac{T(t)}{p_0^2(t) f'_{\mathrm{P}}(p_0(t))^2} - \frac{1}{p_0^2(t) f'_{\mathrm{P}}(p_0(t))^2} - \frac{(1 - p_0(t) m_1)^2}{p_1^2(t) f'_{\mathrm{P}}(p_1(t))^2} \right] \\
&= \ln p_0(t) + \frac{\delta}{T^2(t)} \left[\frac{1 - p_0(t) m_1}{p_0^2(t) f'_{\mathrm{P}}(p_0(t))^2} - \frac{p_0^2(t) m_2^2}{f'_{\mathrm{P}} \left(\frac{1 - p_0(t) m_1}{p_0(t) m_2} \right)^2} \right].
\end{aligned} \tag{SI.188}$$

Recall that $p_0 f'_{\mathrm{P}}(p_0) > 0$ and $f'_{\mathrm{P}}(p_0) > 0$ for $0 < p_0 < 1$ by assumption. Therefore, Eq. (SI.188) is well defined provided $f'_{\mathrm{P}}(\frac{1 - p_0 m_1}{p_0 m_2}) \neq 0$. Requiring $0 \leq p_1 \leq 1$ leads to restrict the feasible values of $\ln p_0$ to the interval

$$X := [-\ln(m_1 + m_2), \min(0, -\ln m_1)] \tag{SI.189}$$

where the lower bound is obtained by rearranging Eq. (SI.185) to $p_1 = (p_0^{-1} - m_1)/m_2$, setting $p_1 \leq 1$ and, then, solving for $\ln p_0$, while the upper bound is similarly obtained by setting $p_1 \geq 0$ and, then, solving for $\ln p_0$ while keeping in mind that it must also be that $p_0 \leq 1$. The dynamics in Eq. (SI.188) are then only meaningful under the restriction that $\ln p_0$ is in D, possibly excluding either end point when Eq. (SI.188) is undefined at those points for the given f_{P}.

3.6.1 Equilibria and stability in the minimal model

Let

$$Q(p_0) := \frac{1 - p_0 m_1}{p_0^2 f'_{\mathrm{P}}(p_0)^2} - \frac{p_0^2 m_2^2}{f'_{\mathrm{P}}(\frac{1-p_0 m_1}{p_0 m_2})^2} \tag{SI.190}$$

be a measure for the change in $\ln p_0$. This function corresponds to the quantity in brackets in the last line of Eq. (SI.188). As δ and T are always positive, any equilibrium $\ln p_0^* \in X$ of Eq. (SI.188) is a solution of the equation $Q(p_0) = 0$. As the quantity $\Delta \ln p_0(t) := \ln p_0(t+1) - \ln p_0(t)$ is directly proportional to $Q(p_0(t))$ and selection is weak ($0 < \delta \ll 1$), equilibria of Eq. (SI.188) and their stability can be visualized by plotting $Q(p_0)$ against p_0 as in Fig. SI.3. This shows that some functions $f_{\mathrm{P}}(p)$ in $\mathscr{F}_+^p$ have a single stable equilibrium, e.g. the identity function, $\exp(p)$, $\sin(p)$ or $-\ln(1-p)$. Yet other functions in $\mathscr{F}_+^p$ can lead to bistability of equilibria with ageing in survival, e.g. $f_{\mathrm{P}}(p) = p^{\frac{2}{5}}$, which reveals that, although all equilibria imply ageing in survival, some may be unstable. As for functions in $\mathscr{F}_-^p$, they can imply both an unstable equilibrium with negative ageing and a stable equilibrium with ageing, e.g. $f_{\mathrm{P}}(p)$ is $\arctan^{\frac{1}{10}}(p)$ or $\sqrt{p}/(1+p)$.

Some analytic results are here derived assuming that f_{P} is either linear or convex, i.e. $f''_{\mathrm{P}}(p) \geq 0$ for $0 < p < 1$ (possibly including either end point when possible), which implies that $f_{\mathrm{P}} \in \mathscr{F}_+^p$. Let Υ be the map $\Upsilon(\ln p_0(t)) = \ln p_0(t+1)$ in Eq . (SI.188). Differentiating this map, one gets

$$\begin{aligned}\frac{\partial \Upsilon}{\partial \ln p_0} = 1 &- \delta \underbrace{\left(\frac{3p_0^2 m_1^2 - 6p_0 m_1 + 4}{p_0^2 T^3 f'_{\mathrm{P}}(p_0)^2}\right)}_{:=a} - \delta \underbrace{\left(\frac{(2p_0^2 m_1^2 - 6p_0 m_1 + 4) f''_{\mathrm{P}}(p_0)}{p_0 T^3 f'_{\mathrm{P}}(p_0)^3}\right)}_{:=b} \\ &- \delta \underbrace{\left(\frac{2p_0 m_2 \left[T f''_{\mathrm{P}}(\frac{1-p_0 m_1}{p_0 m_2}) + 2p_0 m_2 f'_{\mathrm{P}}(\frac{1-p_0 m_1}{p_0 m_2})\right]}{T^3 f'_{\mathrm{P}}(\frac{1-p_0 m_1}{p_0 m_2})^3}\right)}_{:=c}.\end{aligned} \tag{SI.191}$$

Weak selection (i.e. $0 < \delta \ll 1$) guarantees that the derivative in Eq. (SI.191), where defined, always takes values around 1. In particular, when a, b and c are nonnegative with at least one being positive, one has

$$\left| \frac{\partial \Upsilon}{\partial \ln p_0} \right| < 1. \tag{SI.192}$$

To identify cases in which this inequality holds, let $z := p_0 m_1$. The quantity a in Eq. (SI.191) is always positive, as it is directly proportional to the second degree polynomial $3z^2 - 6z + 4$. This polynomial has positive coefficient of the squared term and discriminant of -12 and, therefore, it is positive for all z and, consequently, for all $\ln p_0 \in X$. The quantity b in Eq. (SI.191) is always nonnegative, as it is 0 for linear f_{P} and directly proportional to the second degree polynomial $2z^2 - 6z + 4$ for convex f_{P}. This polynomial has positive coefficient of the squared term and two roots, 1 and 2, which ensure that the polynomial is nonnegative for $0 < z \leq 1$ and, consequently, for $\ln p_0 \in X$. The quantity c in Eq. (SI.191), if well defined, is always positive because of assumptions about the first and second derivatives of f_{P}. Therefore, any equilibrium in the interior of X is linearly stable when f_{P} is either linear or convex. When f_{P} is defined also in the end points of X and so is the quantity c, e.g. when f_{P} is the identity function, $\exp(p)$ or $\tan(p)$, then Υ is a contraction on X and it admits a unique globally stable equilibrium.

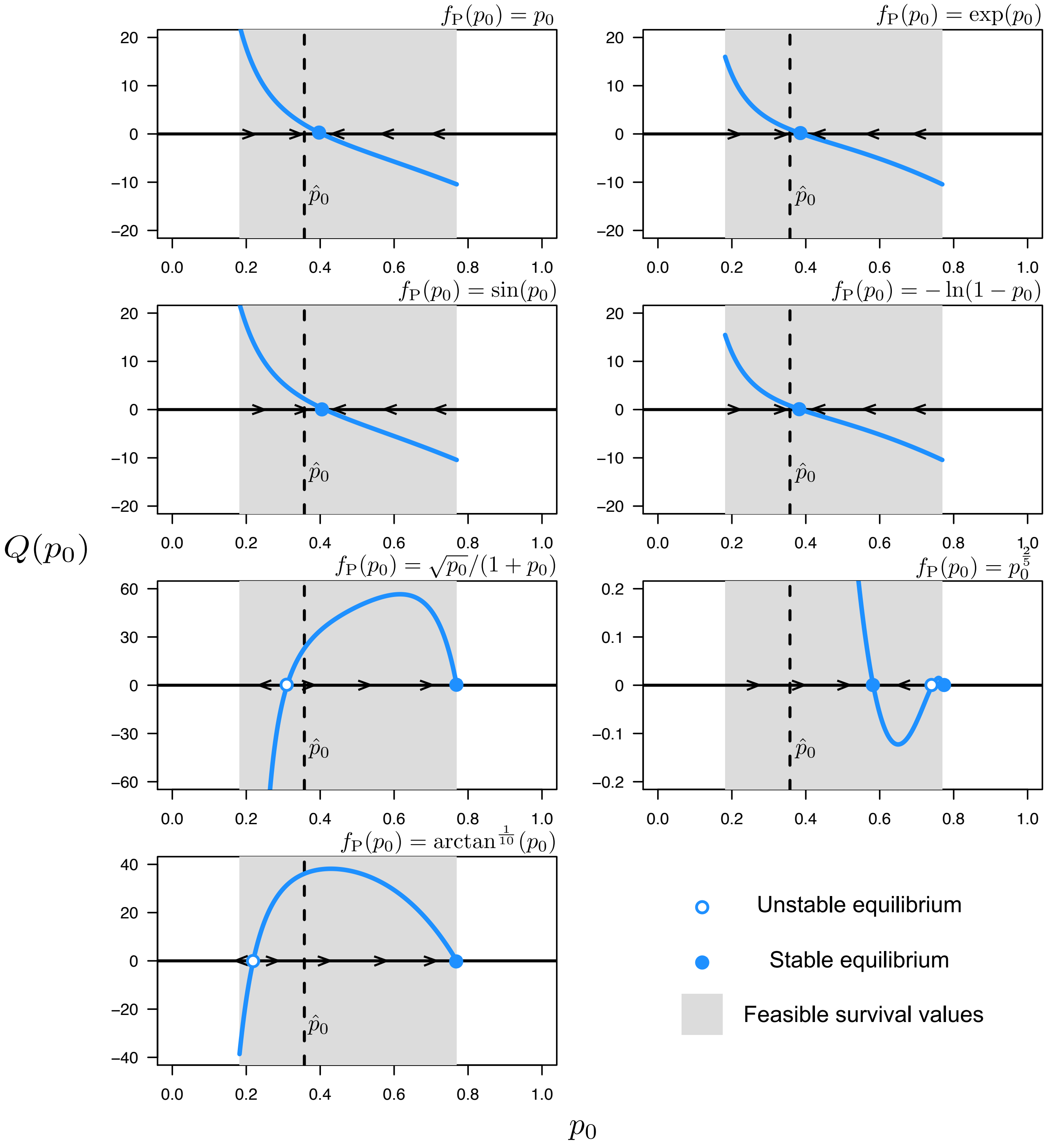

Figure SI.3: **Equilibria and stability in a minimal model of evolution of ageing.** Qualitative evolutionary dynamics are explored in a model with two age classes (ω=2) and reproduction starting from the first (α=1). For different choices of f_{P} in Eqs. (SI.188) and (SI.190), the quantity Q (see main text), which is proportional to the change in $\ln p_0$ over time, is plotted against p_0. The set of values of p_0 so that λ=1 and $0 \leq p_1 \leq 1$ is within the gray area. Fecundities are m_1=1.3 and m_2=4.2. At the right of the vertical dashed line at p_0=$\hat{p}_0$, equilibria show ageing in survival, while at the left of this line there are equilibria with negative ageing.

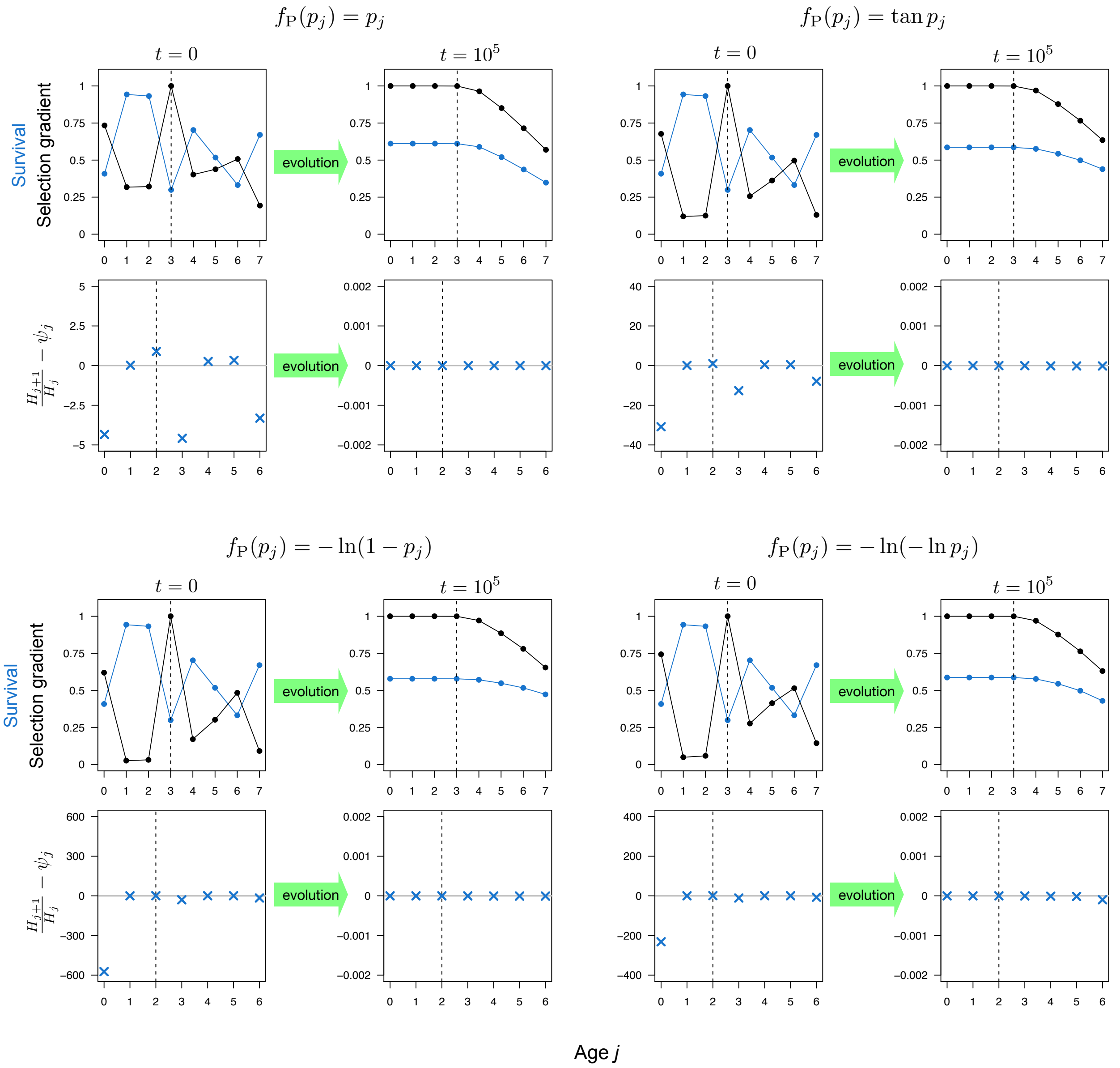

Figure SI.4: **Evolution of survival: numerical solutions.** The results of survival evolution after 10^5 time steps of the dynamics in Eq. (SI.33) imposing a maximum age ω are reported for four different functions $f_\mathrm{P} \in \mathscr{F}_+^p$, see Section 3.3. Functions in this set lead to equilibrium life histories that show ageing in survival, see Section 3.4. The parameter δ (additive genetic variance) in Eq. (SI.33) was set equal to 0.001. Initial survival and fecundities were those in Fig. 1 in the main text. The vertical dashed line indicates the beginning of reproduction. For visualisation, reported selection gradients are scaled by the maximum value they take. The vertical dashed line marks the age of first reproduction. To understand how far away the life history is from a possible equilibrium, the value of $\frac{H_{j+1}}{H_j} - \psi_j$, $j = 0, \ldots, \omega - 2$, was computed. This quantity must be zero at equilibrium, see main text and Eq. (SI.138). In these numerical solutions, life histories eventually approach an equilibrium in which ageing in survival is observed for all four choices of f_P.

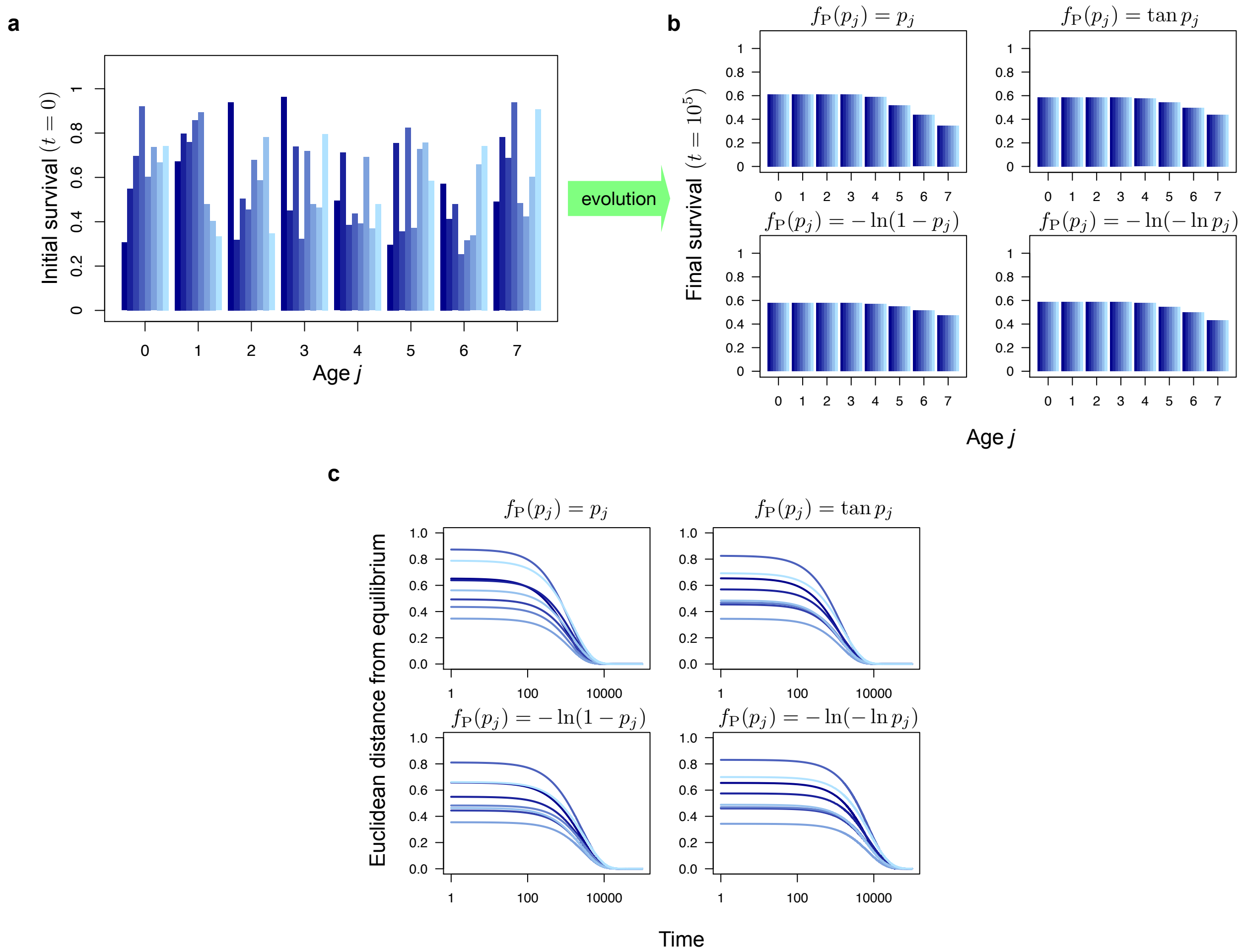

Figure SI.5: **Possible global stability of ageing in survival.** Equilibrium survival levels are estimated under the hypothesis of uniqueness for each of the four functions $f_P \in \mathscr{F}_+^p$ in Fig. SI.4 by numerically iterating for 10^5 time steps the dynamics in Eq. (SI.33) imposing a maximum age ω. **a** Survival for eight different life histories encoded in different color shades. All life histories share the same fecundity as in Fig. 1 of the main text. **b** These eight life histories are used as initial values of the evolutionary dynamics in Eq. (SI.33) under four transformations $f_P \in \mathscr{F}_+^p$ of survival. These transformations lead to any equilibrium life history to show ageing in survival, see Section 3.4. The parameter δ (additive genetic variance) in Eq. (SI.33) is set equal to 0.001. Iterations were stopped after 10^5 time steps. At this point, all evolutionary trajectories have approached the same state. **c** The Euclidean distance of survival from equilibrium, as estimated from numerical solutions in Fig. SI.4, is reported as a function of time, vanishes in all cases regardless of initial conditions suggesting a possible global stability of the estimated equilibria.

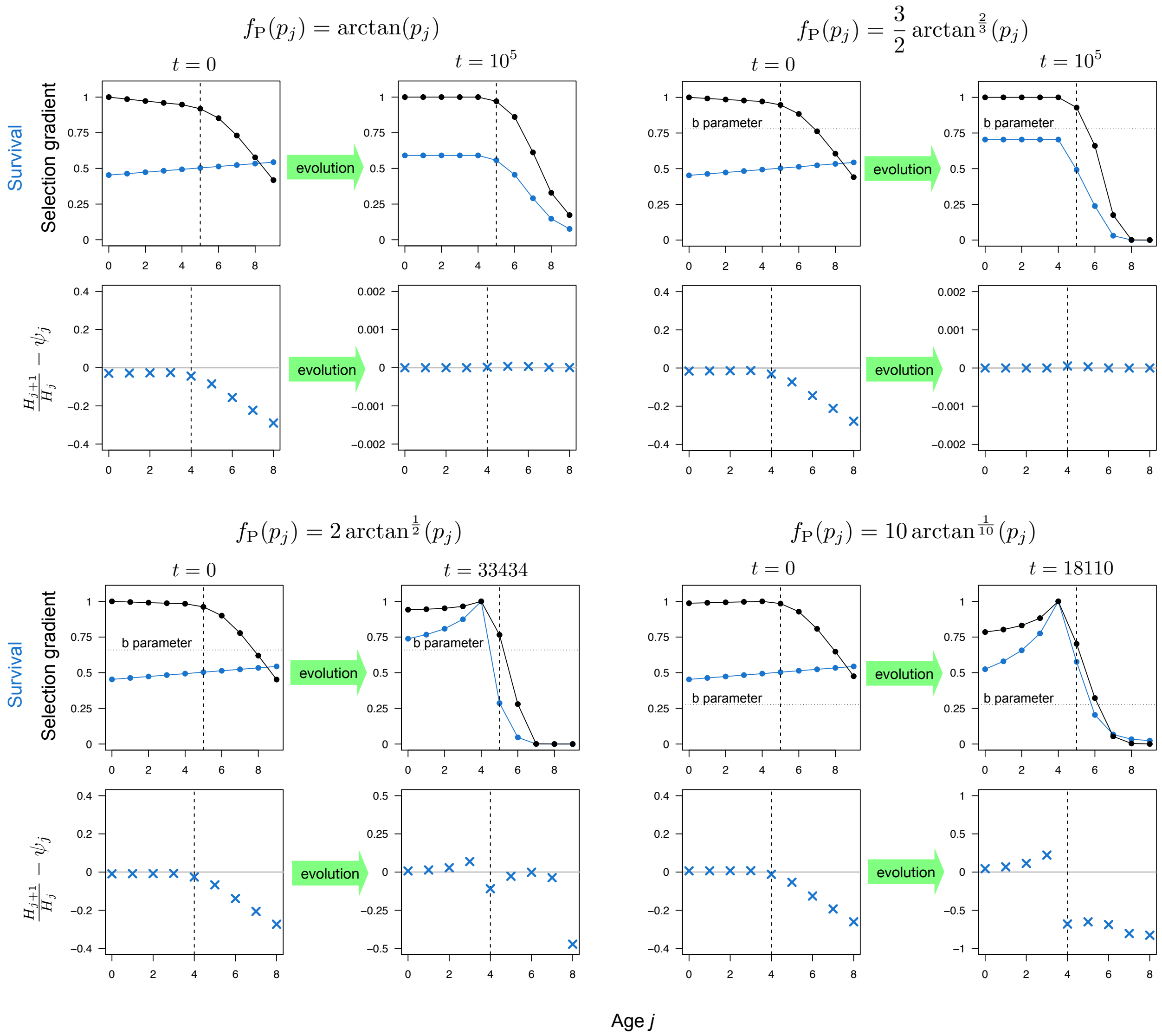

Figure SI.6: **From stability to instability.** For the function $f_P(p) = n \arctan^{\frac{1}{n}}(p) \in \mathscr{F}^p_-$, the dynamics in Eq. (SI.33) imposing a maximum age ω are numerically iterated either up to 10^5 time steps or up to just before survival at some age exceeds unity. Depending on the parameter value n, the quantity b (see Section 3.3) takes different values in the interval between 0 and 1. Functions in $\mathscr{F}^p_-$ lead to different equilibrium life histories, which may show ageing in survival (a stable equilibrium) or not (an unstable equilibrium), see Section 3.4. Here, the cases $n = 1, 1.5, 2, 10$ are considered. The parameter δ (additive genetic variance) in Eq. (SI.33) is set equal to 0.001. Initial survival, equal in all four panels, shows a pattern of negative ageing. Fecundities in all four panels are $m_1 = 0$, $m_2 = 0$, $m_3 = 0$, $m_4 = 0$, $m_5 = 0.8$, $m_6 = 5$, $m_7 = 20$, $m_8 = 50$, $m_9 = 100$, $m_{10} = 500$. For visualisation, reported selection gradients are scaled by the maximum value they take. The vertical dashed line marks the age of first reproduction, while the horizontal dotted line indicates the value of the b parameter for the given transformation of survival $f_P \in \mathscr{F}^p_-$, see Section. (3.3.2). When survival takes values greater than b, which is more likely as b gets closer to 0, eventually the dynamics lead survival to values greater than 1.

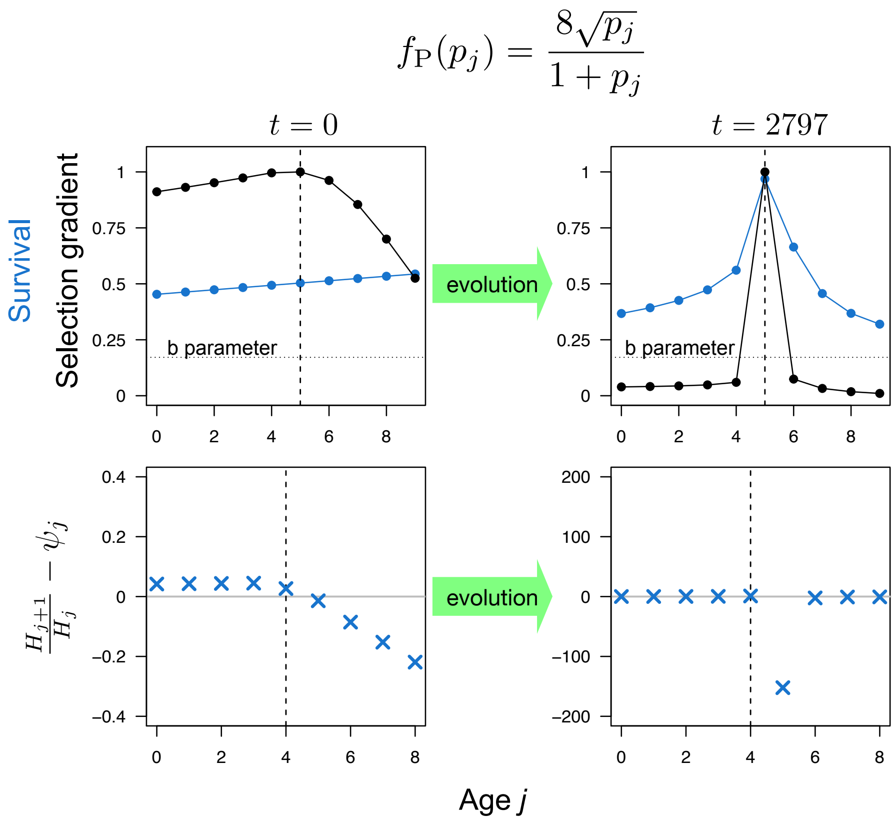

Figure SI.7: **Instability of negative ageing in survival.** A specific function $f_P \in \mathscr{F}^p_-$ is chosen so that the parameter b is much smaller than 1, see Section 3.3. For this choice of f_P, the dynamics in Eq. (SI.33) are numerically iterated imposing a maximum age ω up to either closely approaching an equilibrium or just before survival at some age exceeds unity. The parameter δ (additive genetic variance) in Eq. (SI.33) is set equal to 0.001. Initial survival has a pattern of negative ageing and survival at all ages is greater in value than b. As the quantity $\frac{H_{j+1}}{H_j} - \psi_j$, $j=0,\dots,\omega-2$ shows, initial survival is not far from a possible equilibrium. Fecundities are the same as in Fig. SI.6. For visualisation, reported selection gradients are scaled by the maximum value they take. The vertical dashed line marks the age of first reproduction, while the horizontal dotted line indicates the value of the b parameter for the given transformation $f_P \in \mathscr{F}^p_-$ of survival, see Section 3.3.2. Life history in the population eventually loses the initial pattern of negative ageing and tends to ageing in survival before reaching a point ($t=2797$) after which survival at age 5 exceeds 1. This event shows that for some choice of f_P the dynamics of survival may not be well defined for all times t.

3.7 Boundedness

Here, the problem of dynamics that lead to survival greater than 1 is discussed. Suppose $f_{\mathrm{P}} \in \mathscr{F}^p_+$. Then $p^*_{j+1} = p^*_j$, $j = 0, ..., \alpha - 2$, and $p^*_{j+1} < p^*_j$, $j = \alpha - 1, \alpha, \ldots$. Setting the constant $m_\alpha \geq 1$ suffice to ensure that, at any such equilibrium, survival is bounded between 0 and 1, as the following consideration shows. Since equilibrium pre-reproductive survival is constant, let $p^*_j = u$, $j = 0, ..., \alpha - 1$, for some positive unknown u. Then, $l^*_\alpha = p^*_0 p^*_1 \cdots p^*_{\alpha-1} = u^\alpha$. By Eq. (SI.2) and $\lambda = 1$, at equilibrium one has that

$$1 = u^\alpha \underbrace{(m_\alpha + p^*_\alpha m_{\alpha+1} + \ldots + p^*_\alpha p^*_{\alpha+1} \cdots p^*_{n-1} m_n + \ldots)}_{:=\gamma}. \tag{SI.193}$$

If a partial sum of the series γ is at least 1, then $\gamma > 1$ and $0 < u < 1$ and, consequently, $0 < p^*_j < 1$, $j = 0, 1, \ldots$. This condition ensures that if an equilibrium exists, it is bounded so that survival is between 0 and 1 at all ages. The condition is trivially satisfied when $m_\alpha \geq 1$. Although no formal argument is offered, it is likely that when equilibrium survival exists and is between 0 and 1 at all ages, so should be transient survival because equilibria are found inside attracting sets (see Section 3.5.5).

Suppose $f_{\mathrm{P}} \in \mathscr{F}^p_-$. It is unclear whether some simple condition can guarantee that equilibria with negative ageing are well bounded. Yet instability of pre-reproductive survival for such equilibria and the repelling nature of their containing set lead to conjecture that survival dynamics may exceed unity for $0 < b \ll 1$. If b is only slightly smaller than 1, however, the system may find an equilibrium in which there is ageing and survival is between 0 and 1.

3.8 Numerical solutions

All our results also apply to the dynamics of the finite dimensional system in which we set a maximum age ω instead of working with infinite ages. We explored numerically this simplified system. For $f_{\mathrm{P}} \in \mathscr{F}^p_+$, Fig. SI.4 supports stability of flat pre-reproductive survival and show that the set of life histories with ageing in survival is attracting. Fig. SI.5 is suggestive of the existence of a unique, globally stable equilibrium with ageing in survival for some $f_{\mathrm{P}} \in \mathscr{F}^p_+$ in a model with several age classes. Numerical solutions in Figs. SI.4-SI.5 are consistent with survival evolving within the $(0, 1)$ interval for all ages. End points of evolutionary dynamics are explored in Fig. SI.6. under different partitions of the state space into stable vs. unstable regions induced by different $f_{\mathrm{P}} \in \mathscr{F}^p_-$ functions so that the b parameter, see Section 3.3, goes from being equal to 1 to close to 0. For $f_{\mathrm{P}} \in \mathscr{F}^p_-$, Fig. SI.7 shows that even starting within the set of life histories with negative ageing in survival and not far from an equilibrium, this equilibrium is not approached and dynamics eventually lead survival to exceed 1. We recall that, while numerical iterations are stopped when survival exceeds 1, no mechanism is included in the model to prevent this from happening.

References

[1] Brian Charlesworth. *Evolution in age-structured populations*. Cambridge University Press, Cambridge, 2 edition, 1994.

[2] Ronald A. Fisher. *The Genetical Theory of Natural Selection*. Clarendon Press, Oxford, 1930.

[3] Lamont C. Cole. The population consequences of life history phenomena. *The Quarterly Review of Biology*, 29(2):103–137, 1954. PMID: 13177850.

[4] Russell Lande. A quantitative genetic theory of life history evolution. *Ecology*, 63(3):607–615, 1982.

[5] Brian Charlesworth. Natural selection on multivariate traits in age-structured populations. *Proceedings: Biological Sciences*, 251(1330):47–52, 1993.

[6] Sido D. Mylius and Odo Diekmann. On evolutionarily stable life histories, optimization and the need to be specific about density dependence. *Oikos*, 74(2):218–224, 1995.

[7] Troy Day and Peter D Taylor. Evolutionarily stable versus fitness maximizing life histories under frequency-dependent selection. *Proceedings of the Royal Society of London. Series B: Biological Sciences*, 263:333–338, 1996.

[8] Hal Caswell. *Matrix population models*. Sinauer Associates, 2nd edition edition, 2001.

[9] Jan Kozłowski. Measuring fitness in life-history studies. *Trends in Ecology & Evolution*, 8(84 – 85), 1993.

[10] Jacob A. Moorad. Individual fitness and phenotypic selection in age-structured populations with constant growth rates. *Ecology*, 95(4):1087–1095, 2014.

[11] William D Hamilton. The moulding of senescence by natural selection. *Journal of Theoretical Biology*, 12:12–45, 1966.

[12] William D Hamilton. *Narrow Roads of Gene Land: Evolution of Social Behaviour*. Freeman Spektrum, New York, 1996.

[13] Annette Baudisch. Hamilton's indicators of the force of selection. *Proceedings of the National Academy of Sciences USA*, 102:8263–8268, 2005.

[14] Jacob A. Moorad and Daniel E. L. Promislow. Evolutionary demography and quantitative genetics: age-specific survival as a threshold trait. *Proceedings of the Royal Society B: Biological Sciences*, 278(1702):144–151, 2011.

[15] Daniel E. L. Promislow and Marc Tatar. Mutation and senescence: where genetics and demography meet. *Genetica*, 102(299-314), 1998.

[16] Roger Temam. *Infinite-Dimensional Dynamical Systems in Mechanics and Physics*. Springer-Verlag, New York, 1988.

[17] Linda Partridge and Nick H. Barton. Optimality, mutation and the evolution of ageing. *Nature*, 362(305-311), 1993.
