# Supplementary material for: The selection force weakens with age because ageing evolves and not vice versa
Source: Nat Commun. 2022 Feb 3;13:686. doi: 10.1038/s41467-022-28254-3 (PMC8813929; doi:10.1038/s41467-022-28254-3)
Supplement: Supplementary file 3 — Description of Additional Supplementary Files [file 41467_2022_28254_MOESM3_ESM.pdf]

Description of Additional Supplementary Files

File Name: Supplementary Code 1
Description: This file contains R code embedded in a R Markdown file. The code reproduces Fig. 1 in the main text and all figures in the Supporting Methods file (except Fig.SI.1)
